# Computational analysis of 10,860 phenotypic annotations in individuals with *SCN2A*-related disorders

**DOI:** 10.1038/s41436-021-01120-1

**Published:** 2021-03-17

**Authors:** Katherine Crawford, Julie Xian, Katherine L. Helbig, Peter D. Galer, Shridhar Parthasarathy, David Lewis-Smith, Michael C. Kaufman, Eryn Fitch, Shiva Ganesan, Margaret O’Brien, Veronica Codoni, Colin A. Ellis, Laura J. Conway, Deanne Taylor, Roland Krause, Ingo Helbig

**Affiliations:** 1 Division of Neurology, Children’s Hospital of Philadelphia, Philadelphia, PA USA; 2 Genetic Counseling, Arcadia University, Glenside, PA USA; 3 The Epilepsy NeuroGenetics Initiative (ENGIN), Children’s Hospital of Philadelphia, Philadelphia, PA USA; 4 Department of Biomedical and Health Informatics (DBHi), Children’s Hospital of Philadelphia, Philadelphia, PA USA; 5 Neuroscience Program, University of Pennsylvania, Philadelphia, PA USA; 6 Department of Biology, The College of New Jersey, Ewing Township, NJ USA; 7 Translational and Clinical Research Institute, Newcastle University, Newcastle-upon-Tyne, UK; 8 Royal Victoria Infirmary, Newcastle-upon-Tyne, UK; 9 Luxembourg Centre for Systems Biomedicine, University of Luxembourg, Belvaux, Luxembourg; 10 Department of Neurology, University of Pennsylvania, Philadelphia, PA USA; 11 Department of Pediatrics, University of Pennsylvania Perelman School of Medicine, Philadelphia, PA USA

## Abstract

**Purpose:**

Pathogenic variants in *SCN2A* cause a wide range of neurodevelopmental phenotypes. Reports of genotype–phenotype correlations are often anecdotal, and the available phenotypic data have not been systematically analyzed.

**Methods:**

We extracted phenotypic information from primary descriptions of *SCN2A*-related disorders in the literature between 2001 and 2019, which we coded in Human Phenotype Ontology (HPO) terms. With higher-level phenotype terms inferred by the HPO structure, we assessed the frequencies of clinical features and investigated the association of these features with variant classes and locations within the Na_V_1.2 protein.

**Results:**

We identified 413 unrelated individuals and derived a total of 10,860 HPO terms with 562 unique terms. Protein-truncating variants were associated with autism and behavioral abnormalities. Missense variants were associated with neonatal onset, epileptic spasms, and seizures, regardless of type. Phenotypic similarity was identified in 8/62 recurrent *SCN2A* variants. Three independent principal components accounted for 33% of the phenotypic variance, allowing for separation of gain-of-function versus loss-of-function variants with good performance.

**Conclusion:**

Our work shows that translating clinical features into a computable format using a standardized language allows for quantitative phenotype analysis, mapping the phenotypic landscape of *SCN2A*-related disorders in unprecedented detail and revealing genotype–phenotype correlations along a multidimensional spectrum.

## INTRODUCTION

Over the last decade, more than 100 genetic etiologies have been identified for neurodevelopmental disorders, which include the developmental and epileptic encephalopathies (DEE). The DEE are a group of childhood epilepsies associated with multiple neurological and non-neurological comorbidities that frequently start in the first years of life and are associated with drug-resistant epilepsy^1^. Pathogenic variants in *SCN2A* have emerged as one of the most frequently diagnosed genetic etiologies of DEE^2–5^. The *SCN2A* gene encodes the α-subunit of the neuronally expressed type II voltage-gated sodium channel, also known as Na_V_1.2^6,7^. Both gain-of-function (GoF) and loss-of-function (LoF) mechanisms have been implicated as underlying disease mechanisms, in addition to several complex functional alterations that cannot as easily be categorized^8,9^.

The range of clinical presentation among the *SCN2A*-related disorders is perplexing. Historically, the *SCN2A* gene was identified independently in three distinct phenotypes: benign familial infantile seizures^10,11^, autism spectrum disorders (ASD)^12^, and DEE^13–15^. While these conditions still represent the most well-recognized *SCN2A*-related phenotypes, many clinical presentations overlap, and others have been suggested^8,16^. It has been hypothesized that early-onset epilepsy phenotypes are mainly associated with GoF variants, while later-onset epilepsy and nonepilepsy phenotypes including autism and intellectual disability are associated with LoF variants^6,17^.

Several challenges limit our current understanding of the full phenotypic spectrum and genotype–phenotype correlations in *SCN2A*-related disorders. Clinical descriptions are often limited and may be assessed through different data collection formats. Standardized terminologies, such as the terminology for seizures and epilepsies by the International League Against Epilepsy (ILAE), have changed since the initial description of *SCN2A* as a disease gene in 2001^1,18^. Accordingly, assessing *SCN2A*-related phenotypes using a common framework is paramount to understand the phenotypic spectrum.

Several frameworks have been developed that allow for heterogeneous phenotypic data to be mapped to a common framework. The Human Phenotype Ontology (HPO) is the most frequently used dictionary for harmonization of clinical features (Supplementary Fig. 1)^19^. We have recently demonstrated that the HPO framework can determine clinical similarities between individuals for gene discovery^20^, identify clinical constellations associated with de novo variants^21^, and delineate longitudinal disease phenotypes^22^.

The HPO framework has been used for quantitative phenotypic analysis and high-throughput analysis in other disease areas as well as tools to integrate phenotypic data into diagnostic workflows. For example, mapping electronic medical record (EMR) data to HPO concepts through a novel extraction tool allowed for prioritization of the correct genetic etiology in individuals with monogenic disorders^23^. In addition, HPO terms have been used to annotate the entire corpus of >12 million clinical notes within a single health-care system^24^, demonstrating the utility of this semantically computable vocabulary for large-scale data analysis.

Here, we assessed the clinical phenotypes in 413 unrelated individuals with *SCN2A*-related disorders and mapped phenotypic features to HPO terminology. We harmonized phenotypes across individuals, described the phenotypic landscape, explored phenotypic associations with specific variant types and locations, and examined phenotypic subgroups.

## MATERIALS AND METHODS

### Subject and phenotype extraction

A review of the literature was performed to identify all reported cases of *SCN2A*-related disorders between May 2001 and October 2019. We searched PubMed for studies using the search term “*SCN2A*” and “Nav1.2” and also identified variants using the Human Gene Mutation Database (HGMD) Professional 2020.2^25^. We also included 21 individuals with *SCN2A*-related disorders recruited through the Epilepsy Genetics Research Project at Children’s Hospital of Philadelphia who have not been reported on previously. Only individuals with pathogenic *SCN2A* variants according to the criteria of the American College of Medical Genetics and Genomics/Association for Molecular Pathology (ACMG/AMP) were included^26^. For familial cases, only the proband was included as performed in previous studies^8^.

### Annotation of individuals with HPO terms

We manually assigned HPO terms to all identified individuals with *SCN2A*-related disorders (Supplementary Table 1), using HPO version 1.2 (release format version: 1.2; data version: releases/2018-12-21; downloaded on 2/5/19). HPO term assignment was performed by a genetic counselor with expertise in neurogenetics (K.C.) and reviewed by a senior genetic counselor (K.L.H.) or a senior pediatric neurologist (I.H.).

In addition to “positive” phenotypes (e.g., presence of seizures or autism), we also assigned “negative” phenotypes (e.g., absence of seizures or absence of autism). Negative phenotypes were only coded if absence of the specific phenotype was clearly documented in the literature (Supplementary methods). We used a Compact Internationalized Resource Identifier (CURIE) to refer to HPO terms, i.e., “HP:0001250” (“Seizures”) abbreviates “https://hpo.jax.org/app/browse/term/HP:0001250” in accordance with the Open Biological and Biomedical Ontologies (OBO) Citation and Attribution Policy, as performed previously^21,22^. As a shorthand for negative phenotypes, we coded the absence of “Neurodevelopmental delay” (HP:0012758) as “No neurodevelopmental delay” (NP:0012758).

### Assignment of GoF versus LoF effects

We used the information collected by Lauxmann and collaborators^9^ to identify *SCN2A* variants that had been previously analyzed functionally. We grouped these variants into GoF or LoF variants. Protein-truncating variants (PTV) in *SCN2A* were added to the LoF group, resulting in a total of 17 GoF missense variants and 57 LoF variants, including PTV and missense variants with demonstrated LoF effect (Supplementary Table 3). All other missense variants, including a variant (p.E1211K) with mixed GoF and LoF effects, were classified as neither GoF nor LoF and were excluded in our analyses based on variant-level functional consequences.

### Propagation of HPO terms

To overcome the heterogeneity in the depth of the HPO terminology, we performed automatic reasoning that added all applicable higher-level HPO terms up to the root of the HPO ontological tree for each individual. This propagation of HPO terms is well established for data harmonization^27^, and we have employed this method in our prior work^20–22^. The initially assigned HPO terms were referred to as “base” terms, while the expanded HPO terms were called “propagated” terms. The information content (IC) of each HPO term was defined as -log_2_(*f*), where *f* is the frequency of each HPO term in either the base or propagated data set.

For harmonization of negative HPO terms, we applied a reasoning method similar to propagation of positive HPO terms. However, in contrast to positive HPO terms, the inherent logic within negative HPO terms required an extension from general terms to more specific terms for absent phenotypes. Due to the size of the HPO tree, applying downward propagation resulted in a large number of negative term annotations. Therefore, we applied a “pruning” technique to remove redundant negative terms that cut the HPO tree at branches where no further information was obtained by propagation to more specific phenotypic terms (Supplementary methods; Supplementary Fig. 2).

### Association analysis for variant class and variant location

We performed three separate analyses on the propagated HPO data set, including an association between PTV and missense variants and associations with topological domains within the Na_V_1.2 channel, including all domains and segments. For the association of domains and segments, collectively referred to as “locations,” only missense variants were included. Association analyses for variant class and variant location were performed for all HPO terms in the propagated data set.

### Phenotypic similarity analysis

To assess whether phenotypes in specific subgroups of individuals were more similar than expected by chance, we performed a phenotypic similarity analysis as previously described^20,21^, a method based on that initially introduced by Resnik^28^. In brief, the similarity of a pair of terms is defined as the IC of their most informative common ancestor (MICA), i.e., the term encompassing them both that is least frequently encountered in the propagated HPO data set. The overall similarity of two individuals is derived from summation of the maximum similarities of all pairwise combinations of their respective sets of terms (Supplementary methods; Supplementary Fig. 3). The null distribution of similarity scores for a group of *n* individuals was assessed by randomly selecting and calculating the median similarity of *n* individuals from the overall cohort, with 100,000 permutations as previously reported^20,21^. Exact *p* values for the observed similarities for all groups with *n* group members were determined based on the null distribution of similarity scores.

### Logistic principal component analysis for dimensionality reduction

Given the complexity of the phenotypic data set, we applied dimensionality reduction techniques to compress the highly dimensional HPO data set onto a lower-dimensional feature subspace with the goal of maintaining most of the relevant information. Given that HPO terms were coded either as “assigned” or “not assigned,” we used a logistic principal component analysis (PCA)^29^, coding the presence of an HPO term as 1 and lack of an HPO term for an individual as 0. We assessed the variance explained by individual principal components using a scree plot and generated receiver-operating characteristic curves (ROC) to determine optimal cutoffs to distinguish between broader phenotypic groups and GoF versus LoF variants. ROC performance was measured and interpreted using the area under the curve (AUC) diagnostic. An AUC of 0.7 to 0.8 was considered acceptable performance, 0.8 to 0.9 was considered good performance, and >0.9 was considered great performance.

### Statistical analysis

All statistical analyses were performed using the R computational framework^30^. We performed association analyses using Fisher’s exact test. We corrected for multiple testing using a false discovery rate (FDR) of 10%.

## RESULTS

### Curation of *SCN2A*-related epilepsies

We included 413 unrelated individuals with *SCN2A*-related phenotypes in the final analysis: 392 from published literature and 21 from the Epilepsy Genetic Research Project at Children’s Hospital of Philadelphia. The information in our study was derived from 122 independent publications between May 2001 and October 2019. Individuals were grouped into the following broad phenotypic categories (Supplementary Table 2): DEE (*n* = 255), ASD (*n* = 60), benign familial neonatal–infantile seizures (BFNIS, *n* = 53), other epilepsies (*n* = 27), and atypical phenotypes (*n* = 18). For individuals reported in multiple studies, the most recent phenotypic assessment was used.

### Spectrum of *SCN2A* variants in 413 unrelated individuals

We identified eight types of variants: missense (*n* = 341), nonsense (*n* = 27), frameshift (*n* = 27), splice site (*n* = 13), in-frame deletion (*n* = 2), complex insertion and deletion (*n* = 1), duplication (*n* = 1), and deletion (*n* = 1). Frameshift, nonsense, splice site, and deletion variants were broadly categorized as PTV (*n* = 68). The in-frame deletion, duplication, and complex insertion variants were classified as neither missense nor PTV, and these individuals were excluded from variant analyses. We identified 62 variants in at least two unrelated individuals and 21 variants in at least three individuals (Table 1; full list in Supplementary Table 4). The p.R853Q variant was the most common recurrent variant, found in 18 unrelated individuals.

**Table. 1:** Recurrent variants identified in the cohort.

Recurrent variants	*n*	Broad phenotype (individuals)	Variant class	Location
p.R853Q	18	DEE (16) ASD (2)	Missense	Helical repeat II
p.A263V	14	DEE (8) BFNIS (5) ASD (1)	Missense	Helical repeat I
p.R1882Q	10	DEE (10)	Missense	Cytoplasmic
p.E999K	8	DEE (7) Other epilepsy (1)	Missense	Cytoplasmic
p.L1342P	5	DEE (5)	Missense	Helical repeat III
p.R1319Q	5	BFNIS (3) DEE (2)	Missense	Helical repeat III
p.L1650P	4	Atypical (3) DEE (1)	Missense	Cytoplasmic
p.M1545V	4	DEE (3) Other epilepsy (1)	Missense	Helical repeat IV
p.R1629H	4	DEE (3) BFNIS (1)	Missense	Helical repeat IV
p.V261M	4	DEE (2) BFNIS (2)	Missense	Helical repeat I
p.E1211K	3	DEE (3)	Missense	Helical repeat III
p.E1321K	3	BFNIS (2) ASD (1)	Missense	Cytoplasmic
p.M136I	3	DEE (3)	Missense	Helical repeat I
p.R102*	3	DEE (2) Atypical (1)	Nonsense	Cytoplasmic
p.R1319L	3	DEE (2) Other epilepsy (1)	Missense	Helical repeat III
p.R1435*	3	ASD (2) DEE (1)	Nonsense	Extracellular
p.R36G	3	BFNIS (2) Other epilepsy (1)	Missense	Cytoplasmic
p.R856Q	3	DEE (3)	Missense	Helical repeat II
p.R937C	3	ASD (2) Atypical (1)	Missense	Pore-forming
p.S1336Y	3	DEE (3)	Missense	Cytoplasmic
p.S987I	3	DEE (1) BFNIS (1) Other epilepsy (1)	Missense	Cytoplasmic

Variants identified three or more times in the sample with class and location. Broad phenotype refers to categories described in Supplementary Table 2.

*ASD* autism spectrum disorder, *BFNIS* benign familial neonatal–infantile seizures, *DEE* developmental and epileptic encephalopathies.

### Translation of clinical data into HPO terms

**Fig. 1:**
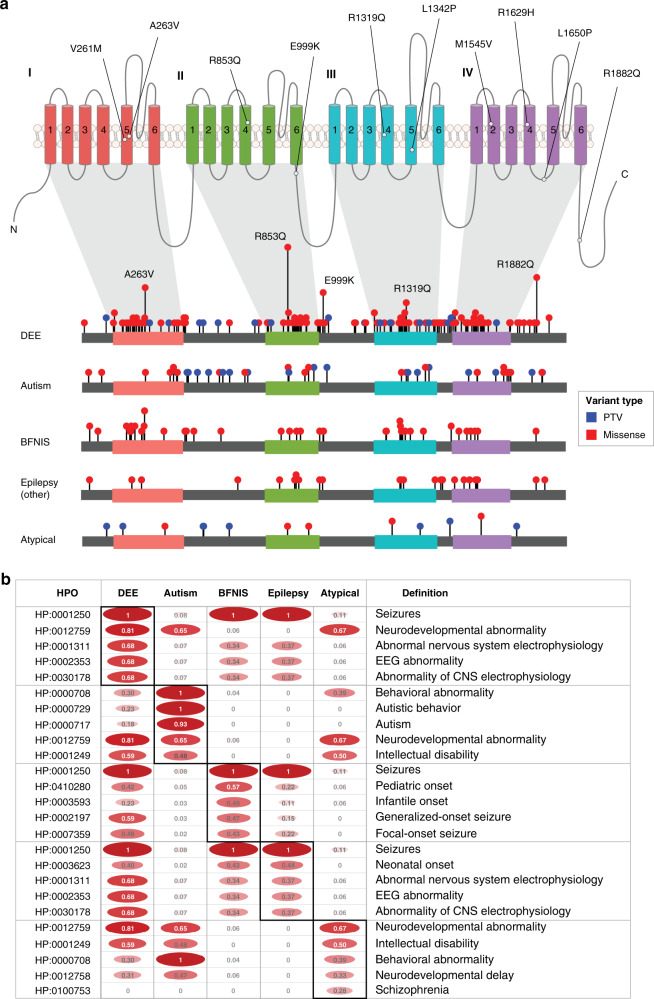
Overview of *SCN2A* variants and associated phenotypic features. (**a**) The Na_V_1.2 channel (above) and gene (below), highlighting a selection of recurrent variants. (**b**) The frequency of phenotypic features within categorized phenotypic subgroups: developmental and epileptic encephalopathy (DEE, *n* = 255), autism (ASD, *n* = 60), benign familial neonatal–infantile seizures (BFNIS, *n* = 53), Other epilepsy (*n* = 27), and atypical *SCN2A*-related phenotypes (*n* = 18). Boxed frequencies indicate the five most frequent Human Phenotype Ontology (HPO) terms within each respective phenotypic subgroup. CNS central nervous system, EEG electroencephalogram, PTV protein-truncating variant.

In the cohort of 413 independent individuals, we encoded a total of 2,935 HPO terms with a median of 6 terms per individual. We inferred higher-level HPO terms and arrived at a total of 10,860 terms (562 unique terms) with a median of 23 HPO terms per individual. IC ranged from 0 to 8.69 with a mean IC of 6.63. We found 359 observed in at least two individuals. Phenotypic frequencies assessed within each phenotypic subgroup are shown in Fig. 1.

### Developing an accurate representation of phenotype frequencies through propagation

Given the heterogeneity with which clinical features in *SCN2A*-related disorders are documented, we analyzed the effect of propagation on HPO term frequencies. We compared the frequencies and the IC of each HPO term before and after propagation (Supplementary Fig. 5). For 30/304 (10%) unique base HPO terms, the difference in frequency in the initially assigned HPO term and adjusted term frequency due to propagation was significant (Supplementary Table 5), indicating that in many individuals these broader phenotypes were implicit in their phenotypic description within the original publication but not explicitly documented. The most prominently affected terms were “Neurodevelopmental abnormality” (HP:0012759), “Seizures” (HP:0001250), and “Interictal EEG abnormality” (HP:0025373).

We separately analyzed phenotypes that were explicitly indicated as being absent in the original literature, which we encoded as negative HPO terms. A total of 475 negative HPO terms were initially assigned in 260/413 individuals (range 1–7 terms, median 2 terms), comprising a total of 18 unique negative base terms. The most frequently assigned negative HPO terms included “No abnormality of brain morphology” (NP:0012443, *f* = 0.31), “No autism” (NP:0000717, *f* = 0.24), and “No seizures” (NP:0001250, *f* = 0.15), using the shorthand for negative HPO terms referred to in “Materials and Methods.” The most common absent or negative HPO terms after propagation included “No hemiballismus” (NP:0100248, *f* = 0.31), “No microcephaly” (NP:0000252, *f* = 0.31), and “No hydrocephalus” (NP:0000238, *f* = 0.31).

### Association of HPO terms with missense variants versus PTVs

Using the harmonized data set of propagated positive and negative HPO terms, we compared the frequencies of HPO terms according to variant class in the 341 individuals with missense variants and the 68 individuals with PTV, shown in Table 2. We found that 35 HPO terms were significant after correction for multiple testing, including 22 HPO terms associated with missense variants and 13 HPO terms associated with PTV. The terms most strongly associated with missense variants were “Neonatal onset” (HP:0003623), “Seizures” (HP:0001250), and “Epileptic spasms” (HP:0011097). The HPO terms most strongly associated with PTV were “Behavioral abnormality” (HP:0000708), “Autism” (HP:0000717), and “Autistic behavior” (HP:0000729). With regard to negative HPO terms, “No intellectual disability” (NP:0001249) was strongly associated with missense variants, whereas “No seizures” (NP:0001250) was strongly associated with PTV. Given the potential complexity of interpreting negative HPO terms, we provided an interpretation of negative phenotypes that accounts for the position of the phenotypic term within the ontological tree (Supplementary Table 6).

**Table. 2:** Phenotypic terms associated with PTV and missense variants (*n* = 409).

HPO term	HPO code	*P* value	Odds ratio (95% CI)	Frequency
*PTV*				
Behavioral abnormality	HP:0000708	4.07×10^-14^	8.61 (4.57–17.01)	0.76
Autism	HP:0000717	1.06×10^-13^	8.16 (4.49–15.13)	0.63
Autistic behavior	HP:0000729	2.31×10^-13^	7.76 (4.26–14.50)	0.68
Stereotypy	HP:0000733	1.30×10^-5^	5.49 (2.45–12.22)	0.24
Sleep disturbance	HP:0002360	1.43×10^-5^	7.04 (2.76–18.32)	0.19
Childhood onset	HP:0011463	0.0004	3.57 (1.71–7.29)	0.25
Delayed speech and language development	HP:0000750	0.0005	3.72 (1.70–7.96)	0.22
Intellectual disability, moderate	HP:0002342	0.0013	3.83 (1.59–8.94)	0.18
Neurodevelopmental abnormality	HP:0012759	0.0023	2.58 (1.35–5.24)	0.79
Short attention span	HP:0000736	0.0047	3.83 (1.38–10.20)	0.13
Hyperactivity	HP:0000752	0.0047	3.83 (1.38–10.20)	0.13
Attention deficit–hyperactivity disorder	HP:0007018	0.0047	3.83 (1.38–10.20)	0.13
Involuntary movements	HP:0004305	0.01	2.65 (1.30–5.25)	0.25
*Missense*				
Neonatal onset	HP:0003623	4.69×10^-14^	Inf (11.88–Inf)	0.40
Seizures	HP:0001250	3.71×10^-13^	8.91 (4.77–16.83)	0.89
Epileptic spasms	HP:0011097	1.44×10^-6^	Inf (4.39–Inf)	0.20
Infantile spasms	HP:0012469	2.62×10^-6^	Inf (4.15–Inf)	0.19
Infantile onset	HP:0003593	3.77×10^-6^	11.44 (2.93–98.45)	0.26
Interictal epileptiform activity	HP:0011182	8.29×10^-6^	3.71 (1.97–7.38)	0.51
Focal-onset seizure	HP:0007359	1.61×10^-5^	4.08 (1.98–9.26)	0.41
Interictal EEG abnormality	HP:0025373	2.85×10^-5^	3.42 (1.83–6.68)	0.51
Abnormal nervous system electrophysiology	HP:0001311	6.00×10^-5^	3.09 (1.70–5.80)	0.55
EEG abnormality	HP:0002353	6.00×10^-5^	3.09 (1.70–5.80)	0.55
Abnormality of CNS electrophysiology	HP:0030178	6.00×10^-5^	3.09 (1.70–5.80)	0.55
Hypsarrhythmia	HP:0002521	0.0001	Inf (2.88–Inf)	0.14
EEG with burst suppression	HP:0010851	0.0002	13.69 (2.27–558.40)	0.17
Generalized-onset seizure	HP:0002197	0.0005	2.77 (1.50–5.34)	0.48
EEG with focal epileptiform discharges	HP:0011185	0.0008	3.20 (1.50–7.61)	0.33
EEG with generalized epileptiform discharges	HP:0011198	0.0014	3.02 (1.46–6.88)	0.34
Multifocal epileptiform discharges	HP:0010841	0.0019	4.27 (1.51–16.70)	0.21
Encephalopathy	HP:0001298	0.0030	2.59 (1.31–5.53)	0.36
Generalized tonic seizures	HP:0010818	0.0030	4.20 (1.48–16.41)	0.21
Epileptic encephalopathy	HP:0200134	0.0065	2.50 (1.26–5.32)	0.35
Focal tonic seizures	HP:0011167	0.0077	5.39 (1.35–46.94)	0.14
Abnormality of the cerebrum	HP:0002060	0.01	2.84 (1.23–7.65)	0.25

All significantly associated terms that remained significant after correction for multiple testing.

*CI* confidence interval, *CNS* central nervous system, *EEG* electroencephalogram, *HPO* Human Phenotype Ontology, *PTV* protein-truncating variants.

### Association of phenotypic features with location of missense variants

We mapped phenotypic features to structural domains in the Na_V_1.2 channel as annotated in Uniprot^31^, analyzing a total of 341 missense variants. Two significant associations remained after correction for multiple testing, both localized to the S5–S6 pore loop domain that confers selective sodium filtering (Supplementary Table 7; variants shown in Supplementary Table 8). Positive association with variants in the S5–S6 pore loop domain was found with “Autism” (HP:0000717, odds ratio [OR] 5.42, 95% confidence interval [CI] 2.39–12.25), while negative association was found with “Seizures” (HP:0001250, OR 0.10, 95% CI 0.04–0.25), showing a similar pattern to PTV. Likewise, missense variants with negative HPO terms showed a strong association between variants in the S5–S6 pore loop and “No seizures” (NP:0001250, OR 9.06, 95% CI 3.33–25.11). There were no significant associations of positive or negative HPO terms with topological domains beyond the S5–S6 pore loop.

### Phenotypic similarity analysis for locations within the NaV1.2 channel and recurrent variants

Phenotypic similarity was nominally significant between individuals with missense variants regardless of location (*p* = 0.01, *n* = 341) and also among individuals with missense variants localized to either the S1 segment (*p* = 0.009, *n* = 18) or domain DIV (*p* = 0.05, *n* = 65). Phenotypic similarity within the group of individuals with missense variants increased when individuals with S5–S6 pore loop missense variants were excluded. The phenotypic similarity in individuals with missense variants in S1 was due to positive associations with “Status epilepticus” (HP:0002133) and “EEG with abnormally slow frequencies” (HP:0011203, Supplementary Fig. 6; variants shown in Supplementary Table 9). The phenotypic similarity in individuals with missense variants in domain DIV was due to positive associations with “Abnormal muscle tone” (HP:0003808), “Polymicrogyria” (HP:0002126), “Spastic tetraplegia” (HP:0002510), “Tremor” (HP:0001337), generalized EEG features (HP:0011199), and a negative association with “Seizures” (HP:0001250, Supplementary Fig. 7; variants shown in Supplementary Table 10).

**Fig. 2:**
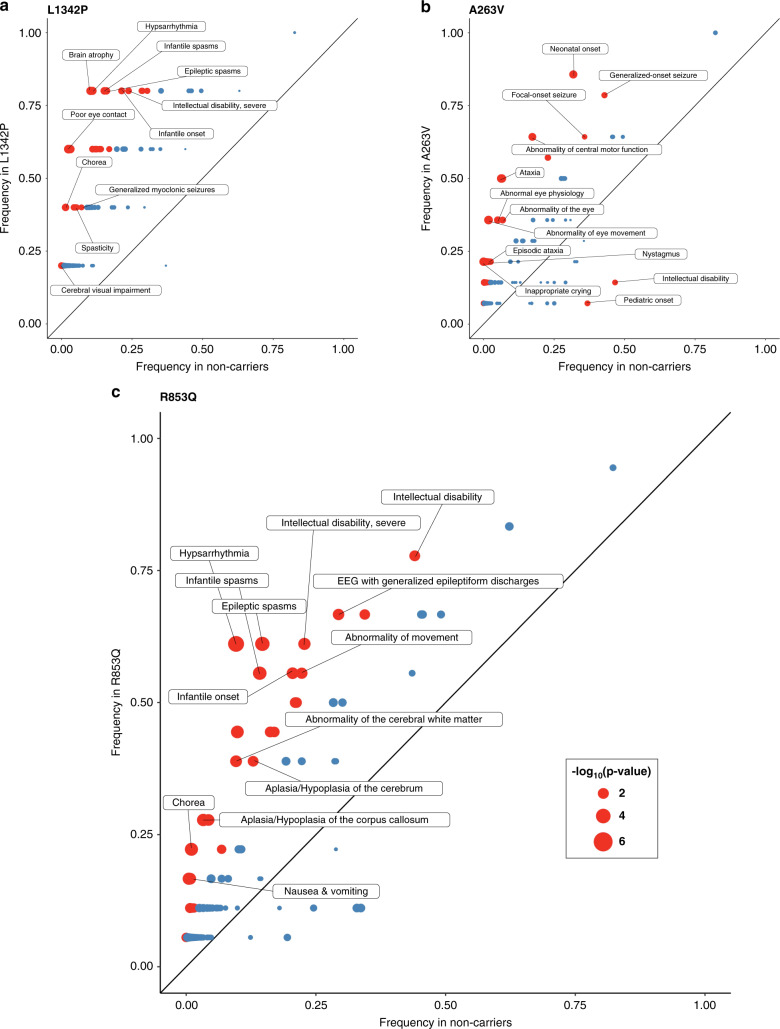
Recurrent variant phenograms. (**a**) Phenogram comparing the frequency of Human Phenotype Ontology (HPO) terms in individuals with variant p.L1342P and the remainder of the cohort. (**b**) Phenogram comparing the frequency of HPO terms in individuals with variant p.A263V and the remainder of the cohort. (**c**) Phenogram comparing the frequency of HPO terms in individuals with variant p.R853Q and the remainder of the cohort. Red points indicate HPO terms with uncorrected *p* values <0.05, blue points indicate HPO terms with uncorrected *p* values ≥0.05. EEG electroencephalogram.

Eight of the 62 recurrent variants and 6 of the 21 recurrent variants identified in three or more individuals were found to have significant phenotypic similarity (Supplementary Table 11). We visualized phenotypic features in the three recurrent variants seen in five or more individuals with *SCN2A*-related disorders (p.L1342P, *p* = 0.0016, *n* = 5; p.A263V, *p* = 0.007, *n* = 14; p.R853Q, *p* = 0.009, *n* = 18) using “phenograms” (Fig. 2) that compare the frequency of HPO terms in individuals with each recurrent variant and the remainder of the cohort^21^. Phenotypic similarity between individuals was not present for all recurrent variants. For example, the recurrent p.R1882Q variant (*p* = 0.29, *n* = 10) did not show overall phenotypic similarity between individuals, even though some individual phenotypic features were associated with this variant.

### PCA

**Fig. 3:**
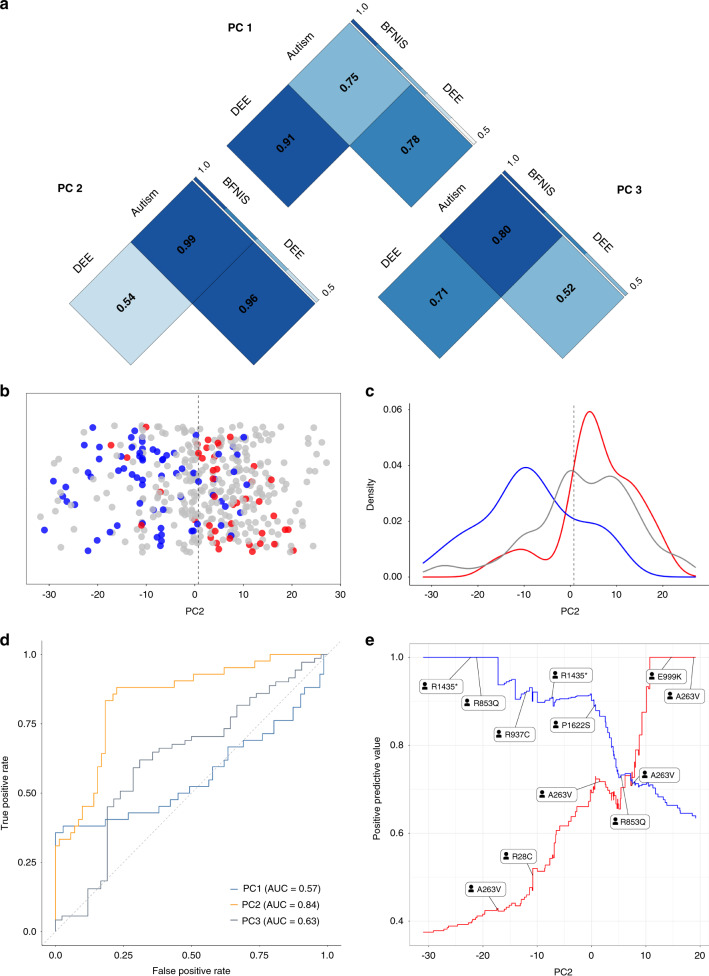
Logistic principal component analysis (PCA) and receiver-operating characteristic curves (ROC) allow for variant function prediction. (**a**) ROC performance measurements (area under the curve [AUC]) for phenotypic subgroup comparisons between developmental and epileptic encephalopathy (DEE), benign familial neonatal–infantile seizures (BFNIS), and autism. A darker shade of blue indicates a higher performance for separating between phenotypic groups. (**b**) The second major principal component (PC2) separates individuals with known loss-of-function (LoF) (blue) and gain-of-function (GoF) (red) variants. (**c**) Density plot of PC2 across all individuals with known LoF (blue), GoF (red), and unmeasured variants (gray). (**d**) ROC for PC2 (yellow) shows higher performance for separating GoF from LoF variants. (**e**) Positive predictive values (PPV) for GoF and LoF variants with PC2 values for individuals with specific variants are highlighted on the graph. Some variants appear twice as phenotypes in individuals with recurrent variants may differ.

The first three components obtained from logistic PCA explained 33.1% of the overall phenotypic variance (PC1 = 15.2%, PC2 = 9.2%, PC3 = 8.7%). We assessed how these components distinguished existing phenotypic groups using ROC curves (Fig. 3) and found that PC1 separates individuals with DEE from other groups (AUC = 0.85, sensitivity = 0.77, specificity = 0.77) and BFNIS from other groups (AUC = 0.86, sensitivity = 0.87, specificity = 0.69). In contrast, PC2 separates individuals with autism from other groups (AUC = 0.96, sensitivity = 0.93, specificity = 0.93). We found acceptable ROC performance for PC3 in separating BFNIS from other groups (AUC = 0.74, sensitivity = 0.62, specificity = 0.83). The ability for the phenotypic dimensions to separate GoF from LoF variants showed good performance with PC2 (AUC = 0.84, sensitivity = 0.83, specificity = 0.82). This suggests that PC2, aligning with a phenotype separation of autism from both DEE and BFNIS, has the potential to predict GoF or LoF variant status based on phenotype. Within our cohort, the top missense variants predicted to be GoF are p.V1326L, p.M1338T, and p.V213D with a positive predictive value of 1 (negative predictive value of 0.63). The top missense variants predicted to be LoF are p.D1487E, p.R922C, and p.I1772M with a positive predictive value of 1 (negative predictive value of 0.38). Given the imbalanced number of GoF and LoF variants, we generated precision-recall curves and F_1_ scores (Supplementary analyses). In this analysis, PC2 remained the strongest principal component to distinguish between GoF and LoF with an F_1_ score of 0.78.

## DISCUSSION

We report our effort to map existing data on *SCN2A*-related disorders onto a common phenotyping framework using HPO terms with methods to harmonize and analyze phenotype data across 562 distinct phenotypic terms. Our study reports the entire clinical literature on a complex neurodevelopmental disease encoded in standardized, computable phenotyping terminology including more than 10,800 phenotypic annotations.

### Using the HPO framework to assess the frequency of clinical features in subgroups

We described the overall frequency of 562 common and rare HPO terms associated with *SCN2A*-related disorders and 296 HPO terms associated with specific variant classes. This analysis provides a significantly more nuanced view of genotype–phenotype correlation than previous studies that relied on qualitative observation of phenotype patterns^8,14,17,32,33^. We found that despite a large degree of heterogeneity, individuals with PTV are significantly less likely than those with missense variants to have seizures^17,33,34^. Overall, individuals with missense variants were more likely to present with early-onset epilepsy with multiple seizure types and abnormalities on electroencephalography.

Missense variants make up the majority of variants reported in individuals with *SCN2A*-related disorders and missense variant location may be important in predicting specific phenotypic features^35–38^. Expanding on prior findings, our systematic phenotypic analysis identified a strong association of variants in the S5–S6 pore loop domain that is strongly associated with autism^17^. This was the only association between topological domains and phenotypes in our analysis.

### Phenotypic similarity analysis

The structured phenotyping language allowed us to identify subgroups and similarities between individuals using established data analysis techniques^20–22^. The advantage of these techniques lies in the formal analysis of a large number of sparse phenotypic annotations that are difficult to compare manually; these techniques allow for phenotypic similarities in subgroups to emerge that are greater than expected by chance. For *SCN2A*-related disorders, this approach revealed several genotype–phenotype correlations that have not previously been recognized. We found that individuals with missense variants, especially when excluding S5–S6 pore loop missense variants; individuals with missense variants in domain DIV; and individuals with missense variants in any of the S1 segments are more phenotypically similar than expected. In addition, several recurrent *SCN2A* variants, most notably p.L1342P, p.A263V, and p.R853Q, showed significant phenotypic similarity. The p.R853Q variant with a homogeneous phenotype consisting of infantile spasms, hypsarrhythmia, and chorea is a prime example of this category. In summary, the computational approach in our study allowed us to demonstrate that individual recurrent variants within the *SCN2A* gene reliably produce distinct phenotypes. While this was previously known for some recurrent variants, our study highlights a much larger degree of genotype–phenotype association for recurrent variants than was previously known.

While we used the Resnik algorithm in our analysis that we applied in previous studies^20,22^, a wide range of other methods to quantify phenotypic similarity have been suggested^39^. We compared the performance of eight additional similarity algorithms from the literature (Supplementary analyses). Most algorithms outperform the conventional Resnik algorithm used in our study, suggesting the potential for deeper insights through optimization of similarity methods. In addition, our study did not fully explore the potential relation between phenotypic features by integrating external data. We exclusively relied on the topology of the HPO graph and were therefore unable to account for phenotypic relationships that cannot be directly inferred through the graph structure of the HPO^40^. Integration of heterogeneous knowledge resources through graph embeddings holds promise to assist phenotypic similarity measurement and patient-level subgrouping. For example, the node similarity measurements in the HPO2Vec+ framework integrating data from resources such as DECIPHER, OMIM, and Orphanet have been shown to outperform traditional similarity algorithms^40^. Accordingly, in addition to optimizing traditional similarity algorithms, vectorized graph representations may be critical in the analysis of large phenotypic data in precision medicine approaches.

### Prediction of variant function through PCA

Using logistic PCA, we found that the three major principal components align with three major phenotypic groups: DEE, autism, and self-limited familial neonatal–infantile seizures, formerly referred to as BFNIS. However, the overall stratification of phenotypes is more complex as these components are independent of each other and dimensionality reduction of phenotypic data enabled by harmonized phenotype data revealed a complex pattern within the *SCN2A*-related disorders that separated these phenotypic groups. We found that PC2 primarily separates between existing GoF and LoF variants with good performance, which can be used to predict variant function. Given that the separation of GoF and LoF may predict treatment response to sodium channel blockers^6,8^, logistic PCA allows for a formal framework to prioritize individuals where treatment with sodium channel blockers may be indicated. Surprisingly, we found that PC1, separating the large DEE group from all other phenotypes, does not separate GoF from LoF variants, possibly due to the high frequency of missense variants in other non-DEE groups, such as BFNIS, indicating that the binary classification of variants into GoF and LoF classes is insufficient to explain the form of epilepsy an individual develops. In summary, our framework to predict variant function based on phenotypes has the potential to refine and complement emerging prediction frameworks based on sequence-based features^41^.

### Analysis of negative phenotypic terms

An analysis of negative phenotypes is particularly pertinent to *SCN2A-*related disorders in which the absence of early-onset seizures or neurodevelopmental abnormalities is thought to be important in distinguishing between disorders with dramatically different prognoses. We found a robust association of the absence of seizures with PTV, mirroring the inverse relationship with the presence of seizures with missense variants. Likewise, we found the absence of autism and developmental delay to be associated with missense variants, reflecting the contribution of the BFNIS group. Taken together, associations with negative phenotypes are important for highlighting critical phenotypic features that are relevant for treatment and counseling. For example, in relation to the overall phenotypic landscape of the *SCN2A*-related disorders, individuals with a novel missense variant are three times more likely not to have autism and almost 20 times more likely to not have any form of intellectual disability.

### Scalability of phenotypic data analysis

Our study exclusively relied on manually annotated information in parallel to our prior studies^20,22^. While manual phenotyping ensures a qualitatively high mapping of phenotypic features, this approach is time-consuming and not scalable. Mapping HPO terms from standardized nomenclatures commonly used in routine clinical care or natural language processing from full-text medical literature or patient notes through tools such as Doc2HPO^42^ may allow for computational phenotyping to become scalable in the future.

### Conclusions

We described the full phenotypic landscape of *SCN2A*-related disorders in 413 individuals using a standardized phenotyping language after systematic data harmonization. This allowed us to analyze the clinical features of *SCN2A*-related disorders in unprecedented detail and identify phenotypic features associated with distinct variant classes and locations. Our results help define subclasses for future precision medicine approaches in *SCN2A*-related disorders and provide a general framework on how HPO terminology can be used to map and understand heterogeneous clinical data in rare disorders.

## Supplementary information

Supplementary material

Supplementary Tables

## Data Availability

Primary data for this analysis are provided in the Supplementary Tables. Computer code for the analysis is freely available at https://github.com/helbig-lab/SCN2A, including the methodology used in prior publications^20–22^.
