# Supplementary material for: Computational analysis of 10,860 phenotypic annotations in individuals with SCN2A-related disorders
Source: Genet Med. 2021 Mar 17;23(7):1263–72. doi: 10.1038/s41436-021-01120-1 (PMC8257493; doi:10.1038/s41436-021-01120-1)
Supplement: Supplementary file 1 — Supplementary material [file 41436_2021_1120_MOESM1_ESM.pdf]

Supplementary Material

- **Supplementary Fig. 1:** Concept of the Human Phenotype Ontology (HPO)
- **Supplementary Table 1**: *SCN2A* primary data table (attached separately)
- **Supplementary methods**
    - Propagation of positive HPO terms
    - Removal of higher-level modifier terms after propagation
    - Coding of HPO terms for distinct epilepsy syndromes
    - Curation of negative HPO terms
    - Propagation and removal of redundant negative phenotypic terms ('pruning') (**Supplementary Fig. 2**)
    - Phenotypic similarity analysis according to Resnik (**Supplementary Fig. 3**)
- **Supplementary analyses**
    - Reporting of *SCN2A* phenotypes across publications
    - Assessing the phenotyping gap (heatmap shown in **Supplementary Fig. 4**)
    - Phenotypic similarity analysis for diagnosis groups
    - Comparison of different similarity algorithms **(Supplementary Fig. 8)**
    - Analysis of precision-recall curves and F1 scores (**Supplementary Fig. 9**)
- **Supplementary Table 2**: Broad phenotypic categories
- **Supplementary Table 3**: Overview of GoF and LoF variants
- **Supplementary Table 4**: List of all 62 recurrent variants
- **Supplementary Table 5**: Change in HPO terms frequencies due to propagation

- **Supplementary Fig. 5**: Distribution of HPO terms before and after propagation
- **Supplementary Table 6:** Negative HPO terms significantly associated with variant type
- **Supplementary Table 7**: HPO terms significantly associated with variant location
- **Supplementary Table 8**: Overview of 27 missense variants in S5-S6
- **Supplementary Fig. 6**: Phenogram for HPO terms in individuals with variants in S1
- **Supplementary Table 9**: Overview of 11 missense variants in S1
- **Supplementary Fig. 7**: Phenogram for HPO terms in individuals with variants in DIV
- **Supplementary Table 10**: Overview of 27 missense variants in DIV
- **Supplementary Table 11**: Phenotypic similarity in individuals with recurrent variants
- **Supplementary Fig. 8:** Phenotypic similarity using different similarity algorithms
- **Supplementary Fig. 9:** Precision-recall curves for PCA analysis
- **Supplementary Table 12**: List of all variants included in study with references

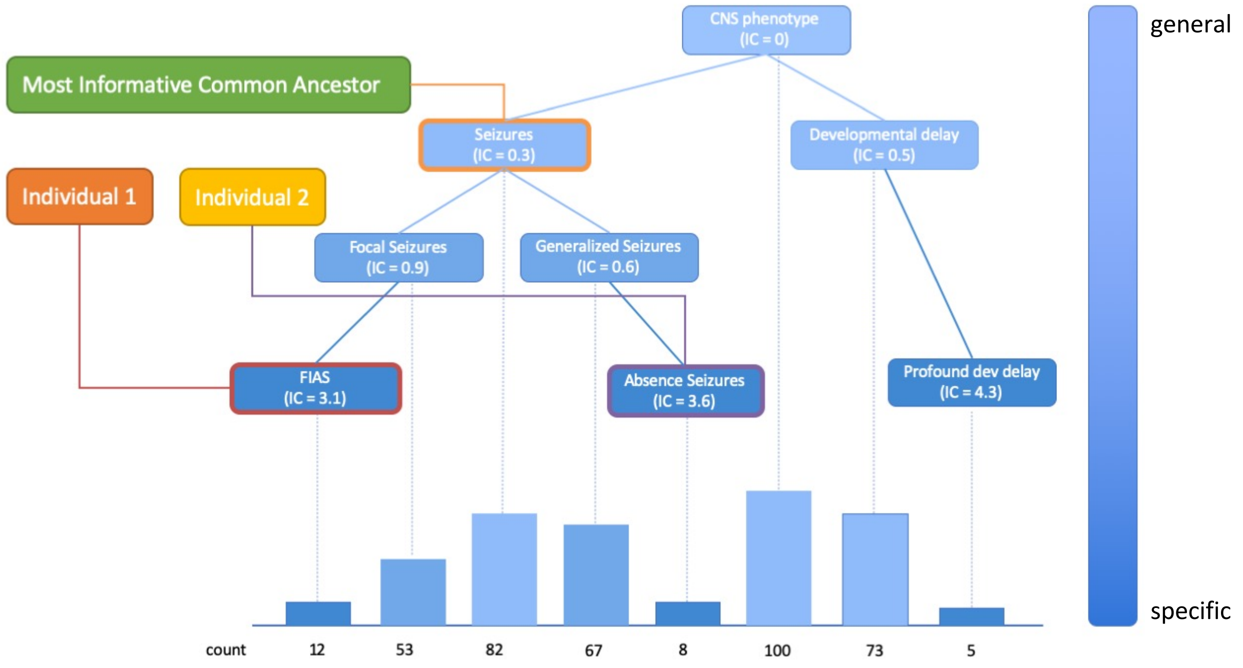

Supplementary Fig. 1 Conceptual framework of the Human Phenotype Ontology (HPO) with respect to seizure terminology (simplified example), demonstrating the relationship between Focal Impaired Awareness Seizures (FIAS) and Absence Seizures. The bottom histogram shows a hypothetical distribution in 100 individuals, which translates into Information Content (IC) for each term, defined as the $-\log_2(f)$ of the frequency f of each HPO term. HPO terms are connected through an 'is_a' relationship with more specific terms as sub terms of more general terms. For each pair of HPO terms, a 'Most Informative Common Ancestor' (MICA) can be determined.

Supplementary methods

Propagation of positive HPO terms

Phenotypic terms can be assigned at various levels of precision, ranging from non-specific high-level phenotypic terms (e.g., 'Seizures', HP:0001250) to very specific phenotypic descriptions (e.g., 'Early-onset absence seizures', HP:0011152). Most often, positive phenotypic terms are annotated from the clinical description at the greatest possible level of detail. Resultingly, the analysis of the direct translation of clinical records into HPO terms underestimates the frequency of higher-level, conceptually broader phenotype terms. However, it can be reasoned that these higher-level terms are important as they may capture large groups of individuals with phenotypically broad but clinically or biologically important similarities.

For example, direct translation of phenotypic descriptions in the source publications assigned 'Seizures' (HP:0001250) to only 145/413 (35%) individuals, but propagation assigned to more specific seizure descriptions in the source publications allowed us to identify that seizures were present in 342/413 (83%) of individuals. A total of 15 unique HPO terms were initially only assigned to one or two individuals but were found in 8–261 individuals after propagation with a median increase of 19-fold. These HPO terms included 'Abnormality of the gastrointestinal tract' (HP:0011024), 'Involuntary movements' (HP:0004305), and 'Abnormal corpus callosum morphology' (HP:0001273).

Removal of higher-level modifier terms after propagation

After propagation, we removed three higher-level modifier HPO terms with no clinical significance ('Onset', HP:0003674; 'Clinical course', HP:0031797; 'Clinical modifier', HP:0012823) as these higher-level terms do not carry meaningful clinical information in contrast to their respective lower-level phenotypic terms (e.g., 'Neonatal onset', HP:0003623).

Coding of HPO terms for distinct epilepsy syndromes

Individuals with documented diagnoses of specific epilepsy syndromes were assigned phenotypic terms that are unequivocally linked to the specific epilepsy syndrome in addition to other features mentioned in the phenotype description. For example, individuals diagnosed with Ohtahara syndrome were assigned the HPO terms 'Neonatal onset' (HP:0003623), 'Epileptic encephalopathy' (HP:0200134), 'Focal tonic seizures' (HP:0011167), and 'EEG with burst suppression' (HP:0010851) given the clinical definition of Ohtahara syndrome. Similarly, we added 'Seizures' (HP:0001250) for individuals in whom 'Epileptic encephalopathy' (HP:0200134) was the only HPO term directly translated from the literature, especially in reports with relatively little phenotypic information where epileptic encephalopathy is regularly used as a shorthand to describe severe, early-onset epilepsies. These cases were classified within the DEE phenotype group for the purpose of analysis. However, in order to avoid overcalling phenotypic features, we assigned discrete phenotypic terms based on specific epilepsy syndromes with caution. We only made such an assignment if the epilepsy syndrome was deemed credible within the context of the overall phenotypic data provided in the respective study.

Curation of negative HPO terms

Coding of explicitly absent phenotypes allows for additional conclusions about the *SCN2A*-related phenotypes that cannot be drawn from positive phenotypes alone. These negative, explicitly absent phenotypic features are valuable as they refer to phenotypes that were unequivocally coded as not present in individuals in the literature, indicating that individuals were specifically assessed for these features and were concluded not to have the specific phenotype at the time of assessment. For example, the BFNIS is not only characterized by early-onset seizures but also due to the self-limiting nature of the seizures and typical developmental outcome, i.e., the absence of 'Neurodevelopmental abnormality' (HP:0012759).

Accordingly, the absence of a positive HPO term does not necessarily mean that the phenotype has been assessed and is explicitly absent. For example, if an individual does not have the assigned HPO term 'EEG abnormality' (HP:0002353), this does not necessarily allow for the conclusion that the EEG is unremarkable, which would be coded as 'No EEG abnormality' (NP:0002353). The absence of this HPO term may also refer to the fact that this phenotype was not assessed. For example, the individual may not have undergone EEG recording or the EEG report may not have been available at the time of phenotyping.

However, the absence of a clinical feature is not always documented in clinical records or published reports unless it is thought to be important at the time. A previous study of a large cohort of individuals with intellectual disability of unselected etiologies was able to use the absence of HPO terms because contributing clinicians were required to complete a standardized

data set of clinical features comprehensively recording each as present or absent.[1] However, such an approach requires data to be provided using structured and research specific clinical record forms, a barrier to data ascertainment and re-evaluation for meta-analysis. To our knowledge, our study is the first to use HPO terminology to harmonize and comprehensively analyze present and absent phenotypes obtained in heterogeneous formats; we included published studies written from different perspectives such as large genetic studies and clinical case reports in the field of epileptology and neurodevelopmental disorders, as well as patient records.

For the principal component analysis, we analyzed explicitly negative HPO terms as separate terms, coding the presence of an explicitly negative term as 1 and lack of an explicitly negative term as 0, e.g., 'Seizures' (HP:0001250) and 'No Seizures' (NP:0001250) were treated as separate terms that could be present or absent (but both could not be present in the same individual).

Propagation and removal of redundant negative phenotypic terms ('pruning')

Negative HPO terms require a different reasoning for data harmonization that we refer to as 'downward propagation'. This is necessary as absent higher-level HPO terms allow for the conclusion that more specific (lower-level) HPO terms are also absent but not *vice versa*. For example, when the absence of 'Global developmental delay' (HP:0001263) was coded, this also implied the absence of more specific HPO terms such as absence of 'Mild global developmental delay' (HP:0011342) and the absence of 'Severe global developmental delay' (HP:0011344). Accordingly, while propagation for positive HPO terms was performed upward in the HPO tree, propagation to harmonize negative HPO terms was performed downward. As with the upward

propagation for positive HPO terms, the frequency of negative terms was calculated after propagation. Due to the size of the ontological tree within the HPO, applying downward propagation resulted in a large number of negative HPO term annotations. For example, the term 'No Seizures' (NP:0001250) has 70 child terms and the term 'No Morphological abnormality of the central nervous system' (NP:0002011) has 695 child terms.

When assessing the full list of negative HPO terms after downward propagation, it becomes apparent that this list of terms includes significant redundancy. While the concept of 'downward propagation' is intuitive, the number of negative HPO terms generated through this method is enormous. When applying the concept of downward propagation to the 475 negative HPO terms assigned in 260 individuals, downward propagation resulted in 910 unique HPO terms with a median of 171 negative HPO terms per individual (range 1–803 HPO terms), many of which were of no distinguishing value as they were present in exactly the same individuals as their parent terms. For example, the negative terms 'No Microcephaly' (NP:0000252), 'No Progressive microcephaly' (NP:0000253), 'No Cessation of head growth' (NP:0004485), 'No Postnatal microcephaly' (NP:0005484), and 'No Congenital microcephaly' (NP:0011451) are simultaneously assigned to 127 individuals but refer to the same general clinical concept. In order to curb the

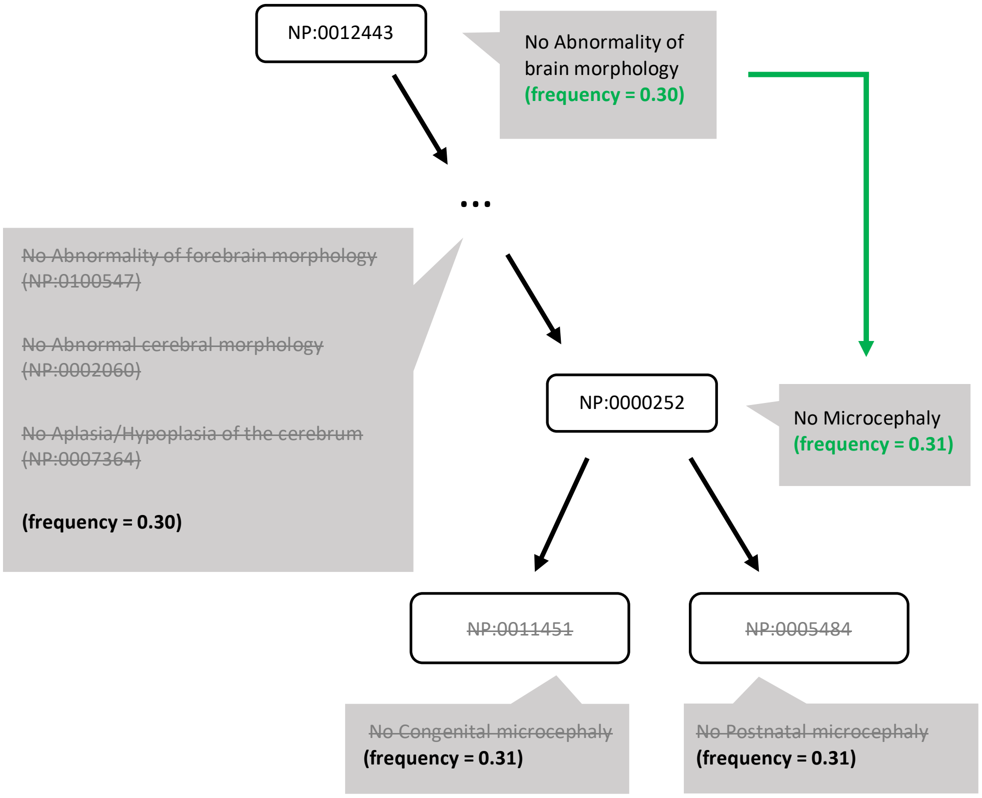

Supplementary Fig. 2 Overview of the concept of pruning negative HPO terms. In order to limit the number of negative HPO terms that arise from the 'downward propagation' of more general HPO terms, the pruning method removes more specific terms if the frequency in the cohort is unchanged.

large number of more specific phenotypes that can be inferred from a high-level, parental phenotype, we applied a method that we refer to as pruning (Supplementary Fig. 2).

The pruning method reduces the number of phenotypic terms by removing branches from the HPO tree when no further information gain is obtained when moving to more specific terms. Our pruning method stops the downward propagation at the conceptually broader, comprehensive HPO term that is equally informative. In the case of the microcephaly concepts mentioned above,

pruning would stop at ‘No Microcephaly’ (NP:0000252) but exclude the child terms, including ‘No Postnatal microcephaly’ (NP:0005484) and ‘No Congenital microcephaly’ (NP:0011451) and their child terms, as these terms are assigned in the same number of individuals and are equally informative. However, it would include the two terms ‘No Abnormality of brain morphology’ (NP:0012443) and ‘No Microcephaly’ (NP:0000252), as the higher-level term ‘No Abnormality of brain morphology’ (NP:0007364) is only coded in 126 individuals, while ‘No Microcephaly’ (NP:0000252) is found in 127 individuals. Pruning would remove the intermediate terms ‘No Abnormality of forebrain morphology’ (NP:0100547), ‘No Abnormal cerebral morphology’ (NP:0002060), and ‘No Aplasia/Hypoplasia of the cerebrum’ (NP:0007364) as these terms were inferred in the 126 individuals from the higher-level term ‘No Abnormality of brain morphology’ (NP:0012443). This is due to the fact that ‘No Microcephaly’ (NP:0000252) was coded in fam273 (PMID 31171384, Patient 15). The pruning method enabled us to reduce the total number of negative, absent HPO terms from 68,079 (910 unique) terms to 856 (22 unique) terms.

Phenotypic similarity analysis according to Resnik

Using the sim_{max} method as previously described,[2,3] we generate a symmetric score for the similarity of two individuals (P_1 and P_2).

A matrix ***S*** holds all HPO terms in individual P_1 (*n* terms as rows) and all HPO terms in individual P_2 (*m* terms as columns). The common parent term of base terms *i* and *j* with the highest information content (IC) is chosen as their most informative common ancestor (MICA), and its IC is provided as the similarity score s_{ij} as shown below in **Supplementary Fig. 3**. The final similarity score of individuals P_1 and P_2 is derived by summing the maximum IC of each column and row of the matrix ***S*** and dividing by two.

$$sim_{max}(P_1, P_2) = \frac{1}{2}\left(\sum_{i=1}^{m} \max_{1 \le j \le n} s_{ij} + \sum_{j=1}^{n} \max_{1 \le i \le m} s_{ji}\right)$$

This can be conceptualized as identifying the most similar feature of P_1 to each feature of P_2 and *vice versa,* then calculating the total similarity of P_1 and P_2 as a function of the specificity of these most similar features.

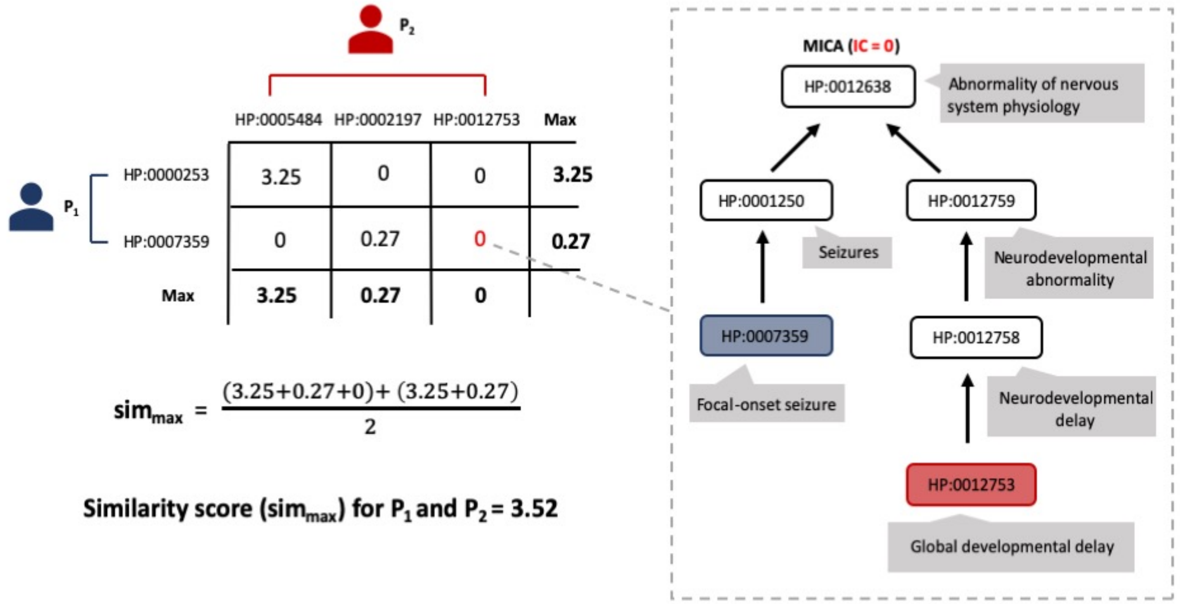

Supplementary Fig. 3 Overview of the phenotypic similarity analysis according to Resnik. A sim score (sim_{max}) is determined by first assessing the Most Informative Common Ancestors (MICA) for all pairs of HPO terms between two individuals. The Information Content (IC) is then summed up across all term pairs and normalized.

Supplementary analyses

Reporting of *SCN2A* phenotypes across publications

In order to compare how phenotypes in *SCN2A*-related disorders have been reported over time and how the reported phenotypes weigh into the overall assessment of the clinical presentations of *SCN2A*-related disorders, we used the concept of Information Content (IC) to compare the description of *SCN2A*-related phenotypes over time. The average IC of publications did not differ between the first case of *SCN2A* published in 2001 through the more recent publications.

We identified that seven original publications reported on patients that were more similar than expected by chance in their phenotypic similarity.[4-9]

Reviewing each publication in detail, this bias was consistent with the focus of the publication to report on specific subgroups, some with unique phenotypic features not identified or reported in other studies. For example, the study by Schwarz focused on children with 'Episodic ataxia' (HP:0002131), a term identified in only eight patients in our study, three of those patients described in the Schwarz paper. Similarly, the study by Vidal focused on patients with significant phenotypic overlap with Rett Syndrome, and the study by Wang focused on individuals with autism. Both Papuc and Kong included detailed findings regarding MRI imaging and EEG testing, respectively. Berecki focused on two recurrent variants (p.R1882Q, p.R853Q) that had similar, severe presentations. The study by Guo focused specifically on patients with autism phenotypes. Accordingly, these publications included patients that were more similar than expected by

chance due to both standardization of phenotypic coding and preselected features examined within their study cohort. For the remaining studies, we did not find evidence that clinical data was reported in a biased way that would result in a higher similarity of the reported phenotypes than expected by chance.

Assessing the 'phenotyping gap'

In an ideal scenario, a phenotyped cohort would be completely assessed for the presence or absence of phenotypic features, e.g., for each phenotype observed in the cohort, every individual would either be coded with the feature being present (positive HPO terms) or explicitly absent (negative HPO terms). Accordingly, in a completely phenotyped cohort the proportion of individuals with positive and negative HPO terms for the same phenotype would add up to 1, and all phenotypic features of interest (potentially including all that are present in at least one individual) would be included.

We applied this concept to assess the phenotyping gap for each HPO term, e.g., the fraction of individuals that are neither assigned a positive or negative HPO term. Merging the propagated frequencies for positive and negative HPO terms identified a total of 204 HPO terms that were assigned to be positive and negative in at least a single individual. We found a median phenotyping gap of 0.81 (e.g., a median of 81% of individuals were neither assigned a positive or negative HPO term). Only four HPO terms have a phenotyping gap of 0.40 or lower, including 'Seizures' (HP:0001250, gap = 0.03), 'Neurodevelopmental abnormality' (HP:0012759, gap = 0.24), 'Abnormality of brain morphology' (HP:0012443, gap = 0.40), and 'Intellectual disability'

(HP:0001249, gap = 0.40). This indicates that authors of previous *SCN2A* publications and clinicians contributing data on our local cases consider these phenotypes to be particularly important. This finding also suggests that the lack of information on any of these four terms, especially ‘Seizures’ (HP:0001250), can reasonably be used to infer that an individual did not in fact have this phenotype. However, a significant proportion of the morbidity in *SCN2A*-related disorders still remains unresolved. By generating a formal framework, we were able to quantify the degree of missing phenotypic information that may inform future phenotyping efforts. We visualized the overall distribution of positive phenotypic base terms using a heatmap as performed previously,[3] shown in **Supplementary Fig. 4**. Most phenotypes are very rare and negative phenotypes are only recorded in a subset of phenotypes to a sufficient degree.

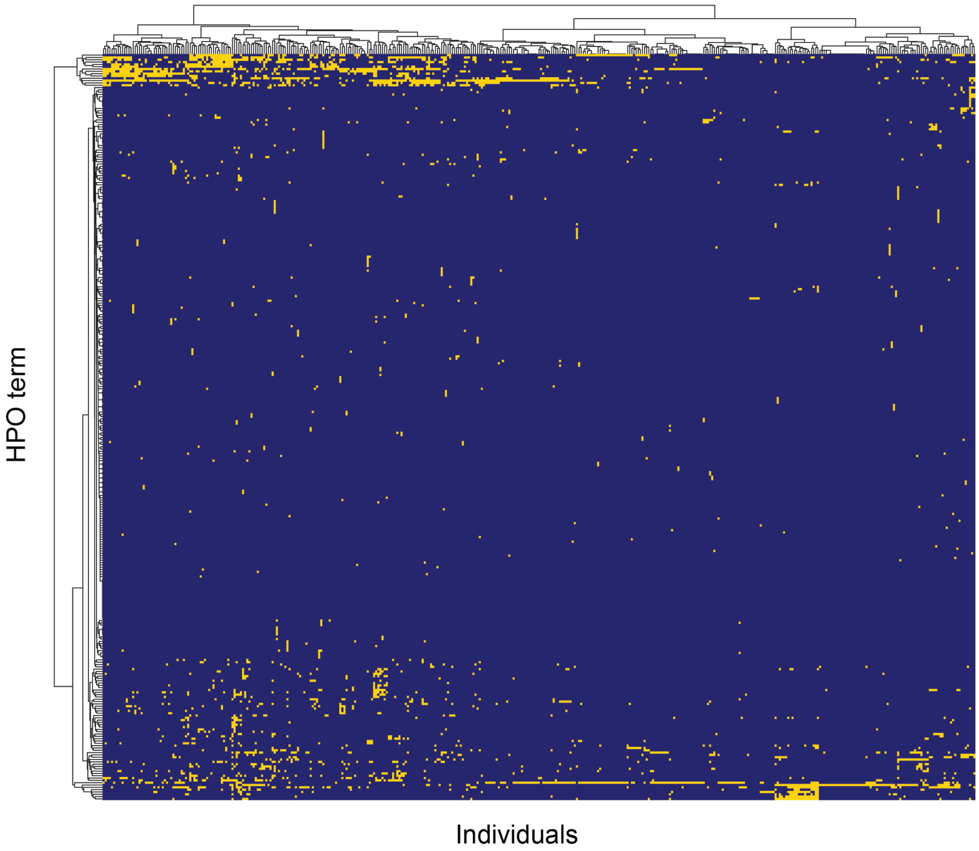

Supplementary Fig. 4 Heatmap of all 413 individuals (x-axis) with all 2,935 positive base HPO terms distributed across 304 unique base terms (y-axis). A yellow dot denotes that the individual has been coded with that term. The plot demonstrates that only a subset of phenotypes is common.

Phenotypic similarity analysis for diagnosis groups

Phenotypic similarity analysis between the five phenotypic groups identified significant ($p < 10^{-6}$) phenotypic similarity between the 254 individuals with DEE but not within any other phenotypic subgroup. This suggests that the phenotypes of individuals assigned to the ASD, BFNIS, other epilepsy group, and atypical *SCN2A* disorders group are coded in an uninformative or heterogeneous way that would not allow these subgroups to be recognized through computational similarity methods. This finding is particularly striking for the BFNIS group that would be considered highly similar within the context of all other *SCN2A*-related disorders, as they stand out clinically because of self-limiting seizures and typical development.[10] However, this result is due to an inherent weakness of the similarity method rather than phenotypic heterogeneity, as MICA-based methods have limitations in picking up the relative absence of additional neurological phenotypes, as is the case in the BFNIS group.

Comparison of different similarity algorithms

For our primary analysis, we used the conventional Resnik measure to assess phenotypic similarity based on the algorithms used in our prior studies.[2,3] However, there are many other similarity measures reported in the literature that can be applied to phenotypic data coded in HPO format.[11] Accordingly, we assessed phenotypic similarity in our cohort across eight additional phenotypic similarity algorithms that – in addition to the conventional Resnik algorithm (#1) – included the algorithm suggested by (#2) Lin,[12] (#3) Jiang and Conrath,[13] (#4) Wang (node-based),[14] (#5) Wang algorithm (edge-based),[14] (#6) Jaccard,[11] (#7) weighted Jaccard,[11] and (#8) Chabalier.[15] We also included an algorithm that we developed in one of our prior study (#9).[16] We compared the performance of all nine algorithms (Resnik algorithm used as the primary algorithm in our study and the eight additional algorithms above) across various categories used in our study including (a) *SCN2A* diagnosis groups, (b) missense variants versus protein-truncating variants, (c) recurrent variants, (d) *SCN2A* domains, (e) *SCN2A* segments, and (f) variants reported within single publications.

We compared the performance of similarity algorithms using a newly developed score that we refer to as the **global similarity score (GS score)**. This score represents the sum of $-\log_{10}(p)$ for all comparisons within a category, divided by the total number of comparisons within this category for normalization (shown in **Supplementary Fig. 8**). In brief, when comparing the same dataset, a similarity algorithm with a higher GS score identifies a higher degree of similarity across all comparisons. Assuming that the ability to detect more similarity reflects true biological signals, algorithms with a higher GS score can be considered superior to algorithms with lower GS scores.

For example, for all recurrent variants within *SCN2A,* the sum of all $-\log_{10}(p)$ divided by all comparisons was 0.69, representing the GS score for recurrent variants obtained by the Resnik algorithm. This score was compared across all algorithms to assess the cumulative significance obtained through these algorithms. In addition, given that the GS score is corrected for the number of comparisons, it can be compared across categories (e.g., diagnosis groups, recurrent variants) to assess how the various algorithms perform across different groups. When comparing the GS score across all algorithms for recurrent variants, the Resnik algorithm used in our study resulted in the lowest GS score, while the weighted Jaccard algorithm (GS score = 1.24) and the Chabalier algorithm (GS score 1.23) performed the best. Accordingly, assuming that an increased GS score reflects a true biological signal, these two algorithms are better suited to detect the phenotypic similarity within recurrent *SCN2A* variants compared to the conventional Resnik algorithm.

When comparing across all categories, the Jaccard algorithm consistently yielded a higher GS score compared to most other algorithms and the conventional Resnik algorithm consistently yielded one of the lowest GS scores. While we used the Resnik algorithm in our study for consistency with our prior studies, the comparison across algorithms suggests that further exploration of similarity algorithms has the potential to be optimized for the recognition of phenotypic similarities.

Analysis of precision-recall curves and F1 scores

The number of gain-of-function (GoF) and loss-of-function variants (LoF) in our study was imbalanced with 42 individuals with GoF and 71 individuals with LoF variants (17 unique GoF and 57 unique LoF variants). This imbalance may complicate the interpretation of Receiver Operating Characteristic curves (ROC curves). In particular, ROC curves can be misleading when data is imbalanced with a high Area Under the ROC curve (AUC) when most or even all items in the smaller group are misclassified.[17] In order to assess whether such a misclassification occurred in our dataset, we generated precision-recall curves (PR curves) and F_1 scores that are considered to be more accurate for imbalanced data. We used the ROCR package in the R Statistical Framework to generate this data (**Supplementary Fig. 9**).[18]

When assessing F_1 scores, PC2 remained the strongest principal component to distinguish between GoF and LoF with an F1 score of 0.78, while PC1 had an F_1 score of 0.53, and PC3 an F_1 score of 0.69. In brief, PC2 still showed acceptable performance in classifying GoF and LoF variants, indicating that the imbalance between datasets did not prominently skew the ROC curve and that these PCs can produce robust classification groupings from our dataset.

Supplementary Table 2. Broad phenotypic categories (n=413)

Broad phenotype	Number of individuals	Year of first description	Number of publications	Median age of onset (IQR), years	Variant type		Recurrent variants (>3 occurrences)		Description of broader phenotype
DEE	255	2004	88	0.12 (0.01–1.00)	Missense<br>PTV	222<br>30	p.R853Q<br>p.R1882Q<br>p.A263V	16<br>10<br>8	Individuals with epileptic encephalopathy
ASD	60	2003	19	1.00 (0.96–1.75)	Missense<br>PTV	32<br>28	None		Individuals diagnosed with autism without diagnosis of DEE
BFNIS	53	2001	22	0.17 (0.01–0.33)	Missense	53	p.A263V<br>p.R1319Q	5<br>3	Individuals with self-limiting neonatal and/or infantile seizures without DEE, ID or DD
Other epilepsies	27	2001	13	0.01 (0.01–0.51)	Missense	26	None		Individuals with epilepsy and phenotypes not compatible with DEE, ASD, or BFNIS
Atypical phenotypes	18	2012	9	0.50 (0.50–0.50)	Missense<br>PTV	8<br>10	p.L1650P	3	Individuals with atypical *SCN2A*-related phenotypes not assigned to other groups

All categories are discrete. Abbreviations: DEE = developmental and epileptic encephalopathy; ASD = autism spectrum disorder; ID = intellectual disability; DD = developmental delay; BFNIS = benign familial neonatal-infantile seizures/epilepsy; PTV = protein-truncating variants

Supplementary Table 3. Overview of 17 gain-of-function and 3 loss-of-function missense variants in *SCN2A*

Variant	Publication (n of individuals)	Overall effect	Functional consequences
p.R28C	Jiang et al. 2013 (1)	GoF	Increased subthreshold current
p.R188W	Ito et al. 2006 (1)<br>Sugawara et al. 2001(1)	GoF	Hyperpolarizing shift of inactivation and decreased slope factor of inactivation; Slowing in time constant for inactivation
p.V208E	Lauxmann et al. 2018 (1)<br>Lemke et al. 2012 (1)	GoF	Hyperpolarizing shift of activation
p.R223Q	Zeng et al. 2018 (1)<br>Berkovic et al. 2004 (1)	GoF	Depolarizing shift of activation and depolarizing shift of inactivation; Increased subthreshold current area
p.V423L	Wolff et al. 2017 (2)	GoF	Increased persistent current
p.T773I	Lauxmann et al. 2018 (1)	GoF	Hyperpolarizing shift of activation; Increased persistent current
p.G899S	Wolff et al. 2017 (1)	LoF	Depolarizing shift of activation
p.K908E	Lauxmann et al. 2018 (1)<br>Wolff et al. 2017 (1)	GoF	Increased current density; Increased subthreshold current
p.R1312T	Shi et al. 2009 (1)	LoF	Hyperpolarizing shift in inactivation and hyperpolarizing shift in activation; Increased slope factor for activation and inactivation; Slowed recovery from fast inactivation; Hyperpolarizing shift in slow inactivation; Use dependence reduced
p.R1319Q	Wolff et al. 2017 (2)<br>Berkovic et al. 2004 (1)	GoF	Depolarizing shift of activation and depolarizing shift of inactivation; Increased subthreshold current area
p.L1330F	Heron et al. 2012 (1)	GoF	Depolarizing shift of inactivation; Decreased slope factor of inactivation curve
p.I1473M	Ogiwara et al. 2009 (1)	GoF	Hyperpolarizing shift of activation
p.Y1589C	Lauxmann et al. 2013 (1)	GoF	Depolarizing shift of inactivation; Accelerated recovery from fast inactivation; Slowed time constant for inactivation; Increased persistent current; Increased subthreshold current area
p.F1597L	Wolff et al. 2017 (1)	GoF	Hyperpolarizing shift of activation; Accelerated recovery from fast inactivation; Slowed time constant for inactivation; Increased slope factor of inactivation curve
p.P1622S	Wolff et al. 2017 (1)	LoF	Hyperpolarizing shift of inactivation; Accelerated time constant for fast inactivation; Increased recovery from fast inactivation; Increased slope factor of the activation curve and inactivation curve
p.R1882G	Schwarz et al. 2016 (2)	GoF	Hyperpolarizing shift of activation
p.L1563V	Heron et al. 2002 (1)	GoF	Neonatal: Depolarizing shift of inactivation; Accelerated recovery from fast inactivation; Slowed time constant for inactivation; Use dependence reduced

			Adult: Hyperpolarizing shift of activation; Decreased slope factor for activation
p.M252V	Liao et al. 2010 (1)	GoF	Neonatal: Increased persistent current; Accelerated recovery from slow inactivation
p.V261M	Kong et al. 2019 (1)<br>Zhang et. al 2017 (1)<br>Wolff et al. 2017 (1)<br>Liao et al. 2010 (1)	GoF	Neonatal: Increased slope factor for activation; Accelerated recovery from fast inactivation and slow inactivation<br><br>Adult: Increased slope factor for activation increased; Accelerated recovery from fast inactivation; Slowed time constant for inactivation; Increased persistent current
p.A263V	Schwarz et al. 2019 (3)<br>Touma et al. 2013 (2)<br>Gorman et al. 2017 (1)<br>Hader et al. 2018 (1)<br>Parrini et al. 2017 (1)<br>Wolff 2017 et al. (1)<br>Nashabat 2019 et al. (1)<br>Halvardson et al. 2016 (1)<br>Liao et al. 2010 (1)<br>Schwarz et al. 2016 (1)<br>Johannesen et al. 2016 (1)	GoF	Neonatal: Increased slope factor for activation curve and inactivation curve; Accelerated recovery from slow inactivation; Slowed time constant for inactivation; Increased persistent current; Increased subthreshold current<br><br>Adult: Depolarizing shift of activation and depolarizing shift of inactivation; Slowed time constant for inactivation; Increased persistent current; Increased subthreshold current

Abbreviations: GoF = gain-of-function; LoF = loss-of-function. PMIDs are listed in **Supplementary Table 1**.

GoF functional elements: Increase in peak current; Acceleration in time constant for activation; Hyperpolarizing shift of activation; Decrease in slope of activation; Slowing in time constant for fast inactivation; Depolarizing shift of fast inactivation; Increase in persistent current; Increase in subthreshold current; Increase in resurgent current; Acceleration of recovery from fast inactivation; Acceleration of recovery from slow inactivation; Decrease of decay in current amplitude in use dependence

LoF functional elements: Decrease in peak current; Slowing in time constant for activation; Depolarizing shift of activation; Increase in slope of activation; Acceleration in time constant of fast inactivation; Hyperpolarizing shift of fast inactivation; Decrease in persistent current; Decrease in subthreshold current; Decrease in resurgent current; Slowing of recovery from fast inactivation; Slowing of recovery from slow inactivation; Increase of decay in current amplitude in use dependence

Supplementary Table 4. List of all 62 recurrent variants in *SCN2A*

Variant	Clinvar ID	Individuals
P.R853Q	191718	18
P.A263V	38843	14
P.R1882Q	193200	10
P.E999K	206981	8
P.L1342P	206996	5
P.R1319Q	12880	5
P.L1650P	870747	4
P.M1545V	207012	4
P.R1629H	207019	4
P.V261M	378927	4
P.E1211K	29886	3
P.E1321K	-	3
P.M136I	-	3
P.R102*	-	3
P.R1319L	379254	3
P.R1435*	419721	3
P.R36G	207035	3
P.R856Q	212125	3
P.R937C	207080	3
P.S1336Y	-	3
P.S987I	206978	3
P.A1773V	520893	2
P.A202V	449147	2
C.476+1G>A	-	2
C.605+1G>T	201282	2
P.C959*	391458	2
P.D343G	661756	2
P.E430G	201303	2
P.F1651C	-	2
P.F928C	-	2
P.G1013*	-	2
P.G1744R	933337	2
P.G211D	-	2
P.I1021YFS*	-	2
P.I1571T	-	2
P.I890M	-	2
P.K1933M	-	2
P.K905N	201341	2
P.K908E	206976	2
P.L881P	425221	2
P.M1323V	375505	2
P.N503KFS*19	-	2
P.Q1521*	-	2

P.Q1531K	-	2
P.R1319W	391627	2
P.R188W	12875	2
P.R223Q	12879	2
P.R379H	-	2
P.R856*	436662	2
P.R937H	432034	2
P.S1758R	405359	2
P.T1420M	-	2
P.T1623N	-	2
P.T227I	-	2
P.V1282F	-	2
P.V1325F	-	2
P.V1528CFS*7	-	2
P.V208E	-	2
P.V213D	-	2
P.V251I	375510	2
P.V423L	207052	2
P.W1398*	-	2

Supplementary Table 5. HPO terms with significant changes in frequency due to propagation

HPO term	HPO code	P-value	Base frequency	Propagated frequency
Neurodevelopmental abnormality	HP:0012759	8.86×10^{-104}	0.002	0.63
Interictal EEG abnormality	HP:0025373	4.54×10^{-67}	0.005	0.47
Generalized-onset seizure	HP:0002197	1.51×10^{-59}	0.01	0.44
EEG abnormality	HP:0002353	9.39×10^{-53}	0.05	0.50
Behavior abnormality	HP:0000708	3.13×10^{-49}	0.01	0.35
Seizures	HP:0001250	8.51×10^{-46}	0.35	0.83
EEG with generalized epileptiform discharges	HP:0011198	1.44×10^{-40}	0.005	0.31
Encephalopathy	HP:0001298	1.70×10^{-40}	0.01	0.33
Intellectual disability	HP:0001249	8.71×10^{-39}	0.07	0.46
Autistic behavior	HP:0000729	6.07×10^{-32}	0.02	0.29
Abnormality of movement	HP:0100022	4.81×10^{-26}	0.01	0.24
Abnormality of brain morphology	HP:0012443	1.35×10^{-24}	0.04	0.29
Aplasia/Hypoplasia of the cerebrum	HP:0007364	2.59×10^{-17}	0.002	0.14
Involuntary movements	HP:0004305	2.46×10^{-16}	0.002	0.13
Epileptic spasms	HP:0011097	1.28×10^{-13}	0.02	0.17
Abnormality of coordination	HP:0011443	2.18×10^{-9}	0.002	0.08
Focal-onset seizure	HP:0007359	3.21×10^{-8}	0.19	0.37
Abnormality of the cerebral white matter	HP:0002500	3.19×10^{-7}	0.02	0.11
Hypertonia	HP:0001276	9.63×10^{-7}	0.02	0.09
Abnormal corpus callosum morphology	HP:0001273	2.83×10^{-5}	0.005	0.05
Abnormality of the gastrointestinal tract	HP:0011024	3.31×10^{-5}	0.002	0.05
Abnormal pattern of respiration	HP:0002793	6.44×10^{-5}	0.002	0.04
Neurological speech impairment	HP:0002167	9.99×10^{-5}	0.005	0.05
Impaired social interactions	HP:0000735	0.0009	0.002	0.03
Abnormal social behavior	HP:0012433	0.0012	0.005	0.04
Abnormal cerebellum morphology	HP:0001317	0.0032	0.002	0.03
Absence seizure	HP:0002121	0.0042	0.02	0.07
Brain atrophy	HP:0012444	0.0083	0.06	0.11
Feeding difficulties	HP:0011968	0.033	0.02	0.05
EEG with focal spike wave	HP:0011197	0.038	0.002	0.02

All significantly associated terms that remained significant after correction for multiple testing.

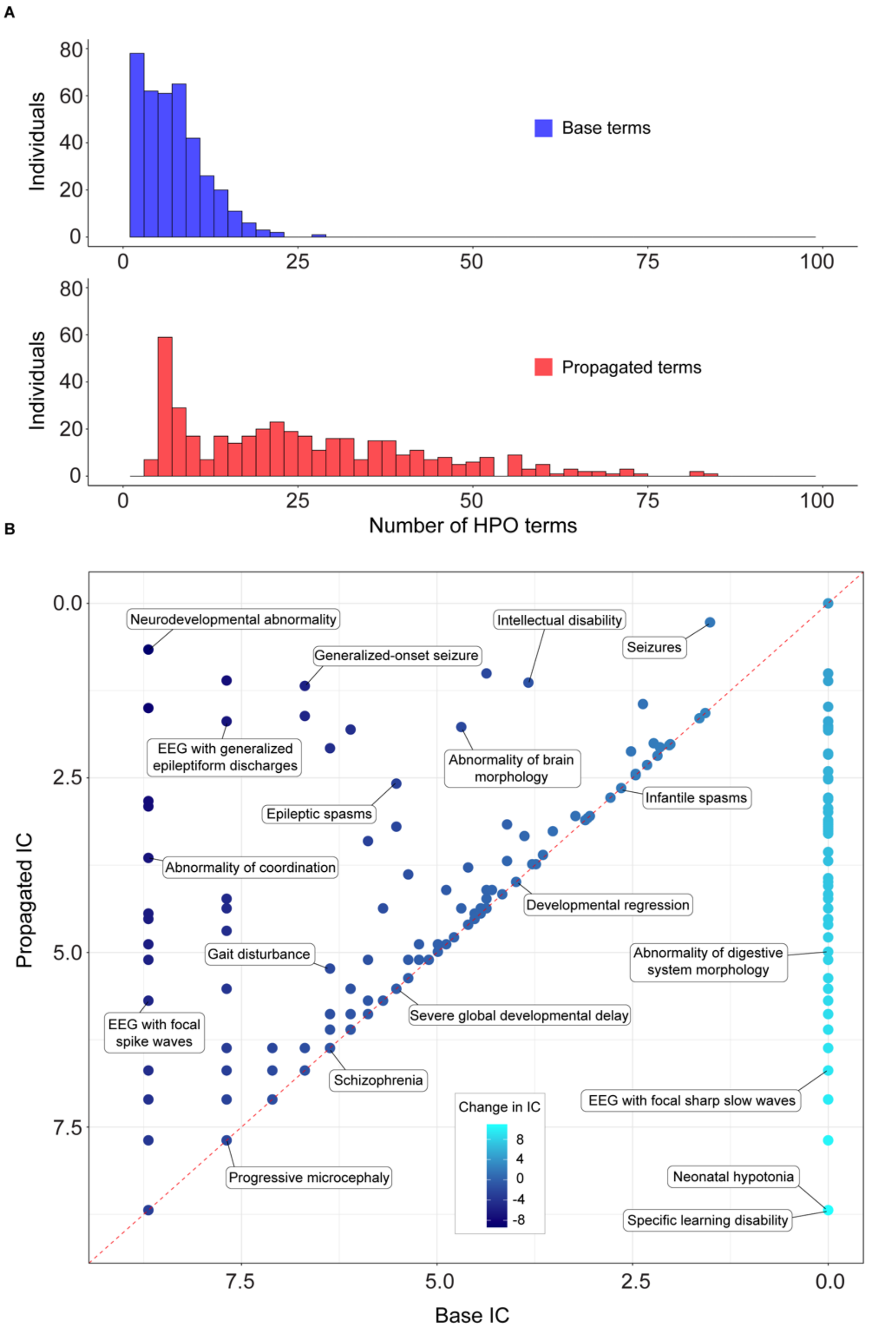

Supplementary Fig. 5 Distribution of HPO terms before and after propagation (**A**) Histograms displaying the number of HPO terms coded to each individual before and after higher-level phenotypic terms were included (i.e., parent terms, 'propagation'). (**B**) Icicle plot showing the change in information content (IC) of HPO terms before and after propagation, reflecting how the increase in frequency makes terms less informative. Terms with base IC of zero only appeared through propagation.

Supplementary Table 6. Negative HPO terms significantly associated with variant type

Negative HPO term (Interpretation)	HPO code	P-value	Odds ratio (95% CI)	Frequency
**PTV**				
No Seizures (*Absence of any type of seizure and epilepsy*)	NP:0001250	1.03×10^{-10}	9.30 (4.45–19.99)	0.61
**Missense**				
No Intellectual disability (*Absence of any type of global intellectual disability, but developmental delays, specific learning disabilities, developmental stagnation and regression may be present, if not resulting in a diagnosis of intellectual disability*)	NP:0001249	1.54×10^{-6}	Inf (4.57–Inf)	0.27
No Developmental regression *(No loss of acquired developmental milestones)*	NP:0002376	5.69×10^{-6}	Inf (4.13–Inf)	0.25
No Global developmental delay *(Either no developmental delay or, if present, developmental delay must be limited to only one domain, regardless of severity)*	NP:0001263	5.81×10^{-6}	Inf (4.03–Inf)	0.25
No Neurodevelopmental abnormality (*Typical development, including absence of developmental delay, intellectual disability, specific learning disabilities, developmental regression and stagnation*)	NP:0012759	5.81×10^{-6}	Inf (4.03–Inf)	0.25
No Absent speech (*Presence of spoken words, but speech delay may be present*)	NP:0001344	0.0001	15.81 (2.56–651.14)	0.25
No Autism (*Absence of autism, but autistic behavior may be present*)	NP:0000717	0.0018	3.22 (1.44–7.95)	0.42

All significantly associated negative terms that remained significant after correction for multiple testing.

Abbreviations: PTV = protein-truncating variants; CI = confidence interval

Supplementary Table 7. HPO terms significantly associated with variant location (n=341)

HPO term	HPO code	Variant location	P-value	Odds ratio (95% CI)	Frequency
**Positive associations**					
Autism	HP:0000717	S5-S6	$1.87x10^{-5}$	5.42 (2.39-12.25)	0.47
**Negative associations**					
Seizures	HP:0001250	S5-S6	$5.55x10^{-7}$	0.11 (0.05-0.27)	0.59

All significantly associated terms that remained significant after correction for multiple testing. Abbreviations: CI = confidence interval

Supplementary Table 8. Overview of 26 missense variants in S5-S6

Variant	Domain	Individuals	Broad phenotype
p.F328V	DI	1	DEE
p.D343G	DI	2	ASD (1)<br>Atypical (1)
p.T365M	DI	1	ASD
p.R379H	DI	2	ASD
p.Q383E	DI	1	DEE
p.L392L	DI	1	ASD
p.T393K	DI	1	DEE
p.R395P	DI	1	ASD
p.T400R	DI	1	DEE
p.G899S	DII	1	DEE
p.Q901E	DII	1	Other epilepsy
p.K905N	DII	2	DEE
p.K905Q	DII	1	DEE
p.K908E	DII	2	DEE (1)<br>BFNIS (1)
p.R922C	DII	1	ASD
p.F928C	DII	2	DEE
p.H930Q	DII	1	DEE
p.R937C	DII	3	ASD (2)<br>Atypical (1)
p.R937H	DII	2	ASD
p.I1281F	DIII	1	DEE
p.K1362M	DIII	1	BFNIS
p.C1386R	DIII	1	DEE
p.K1422E	DIII	1	DEE
p.F1682S	DIV	1	DEE
p.G1744R	DIV	2	ASD
p.G1744E	DIV	1	ASD

Broad phenotype refers to categories described in **Supplementary Table 2.**

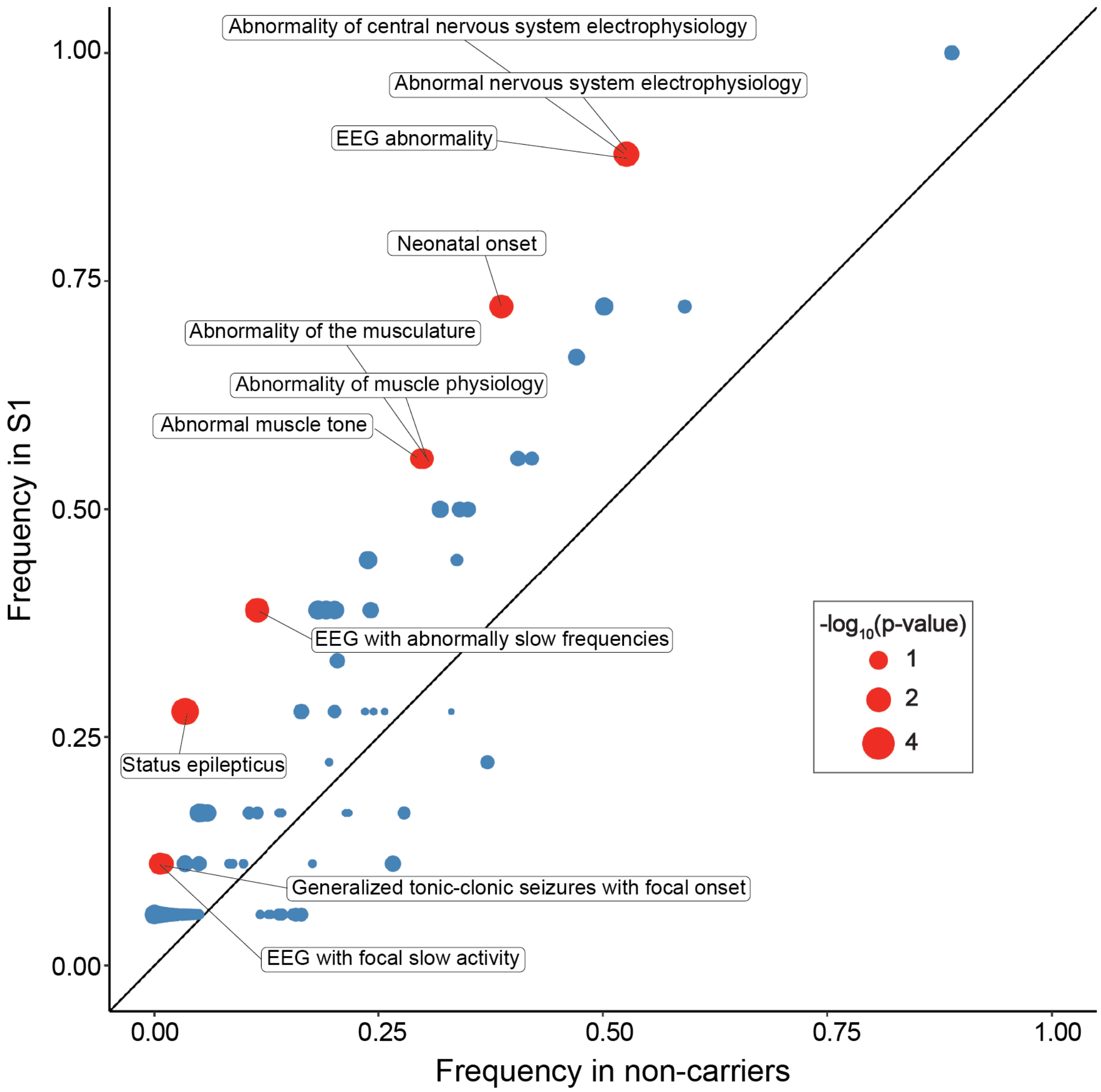

Supplementary Fig. 6 Frequency of phenotypic features in individuals with missense variants in S1 segments compared to the remainder of the cohort.

Supplementary Table 9. Overview of 11 missense variants in segment S1

Variant	Domain	Individuals	Broad phenotype
p.M136I	DI	3	DEE
p.N132K	DI	1	DEE
p.I769T	DII	1	DEE
p.T773I	DII	1	DEE
p.E1211K	DIII	3	DEE
p.G1223R	DIII	1	DEE
p.M1545I	DIV	1	Other epilepsy
p.M1545V	DIV	4	DEE (3)<br>Other epilepsy (1)
p.M1548T	DIV	1	DEE
p.M1548V	DIV	1	DEE
p.S1536R	DIV	1	DEE

Broad phenotype refers to categories described in **Supplementary Table 2.**

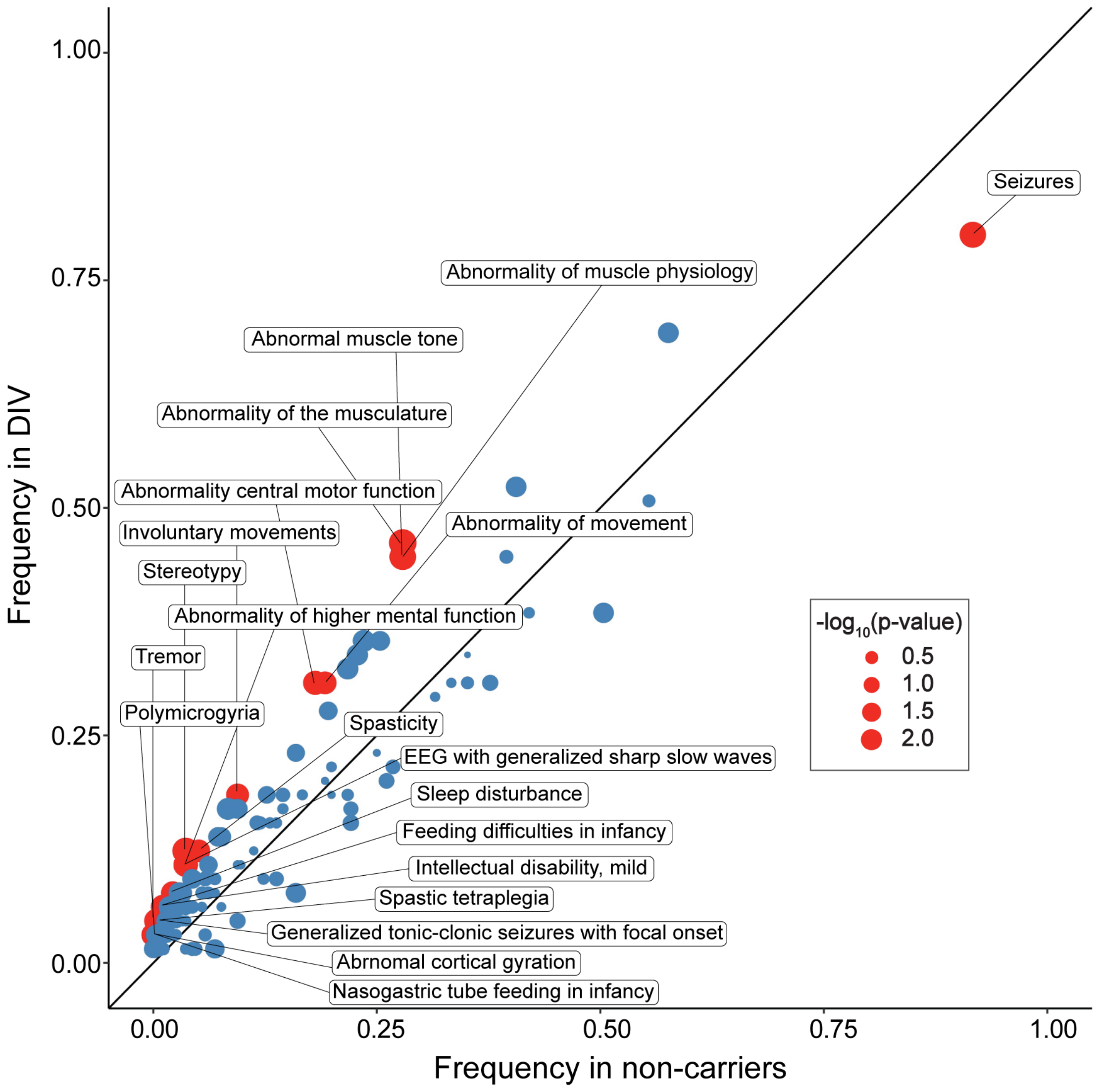

Supplementary Fig. 7 Frequency of phenotypic features in individuals with missense variants in domain DIV compared to the remainder of the cohort.

Supplementary Table 10. Overview of 50 missense variants in domain DIV

Variant	Segment	Individuals	Broad phenotype
p.S1536R	S1	1	DEE
p.M1545I	S1	1	Other epilepsy
p.M1545V	S1	4	DEE (3)<br>Other epilepsy (1)
p.M1548T	S1	1	DEE
p.M1548V	S1	1	DEE
p.I1571T	S2	2	DEE
p.L1563V	S2	1	BFNIS
p.S1584C	S2-S3	1	Other epilepsy
p.Y1589C	S2-S3	1	DEE
p.G1593R	S3	1	DEE
p.W1594R	S3	1	DEE
p.I1596S	S3	1	BFNIS
p.F1597L	S3	1	DEE
p.D1598G	S3	1	DEE
p.V1607A	S3	1	Other epilepsy
p.A1612G	S3-S4	1	BFNIS
p.L1614P	S3-S4	1	DEE
p.P1622S	S3-S4	1	DEE
p.T1623N	S3-S4	2	DEE
p.R1626Q	S4	1	DEE
p.V1627M	S4	1	DEE
p.R1629H	S4	4	DEE (3)<br>BFNIS (1)
p.R1629L	S4	1	DEE
p.R1632K	S4	1	Other epilepsy
p.G1634D	S4	1	ASD
p.G1634V	S4	1	DEE
p.R1635Q	S4	1	ASD
p.I1636M	S4	1	Other epilepsy
p.I1640F	S4	1	Other epilepsy
p.I1640N	S4	1	DEE
p.I1640S	S4	1	DEE

p.K1641N	S4-S5	1	BFNIS
p.L1650P	S4-S5	4	Atypical (3)<br>DEE (1)
p.F1651C	S4-S5	2	DEE
p.A1652P	S4-S5	1	DEE
p.M1654I	S4-S5	1	DEE
p.S1656F	S4-S5	2	DEE
p.L1660W	S5	1	DEE
p.L1665F	S5	1	DEE
p.F1682S	S5-S6	1	DEE
p.G1744E	S5-S6	1	ASD
p.G1744R	S5-S6	2	ASD
p.S1758R	S6	2	ASD
p.L1765P	S6	1	ASD
p.M1770L	S6	1	DEE
p.Y1771H	S6	1	DEE
p.I1772M	S6	1	ASD
p.A1773T	S6	1	ASD
p.A1773V	S6	2	ASD (1)<br>DEE (1)

Broad phenotype refers to categories described in **Supplementary Table 2.**

Supplementary Table 11. HPO term associations in recurrent *SCN2A* variants with nominally significant phenotypic similarity

Variant (sim p-value)	HPO term	HPO code	Group frequency	Cohort frequency	Odds ratio (95% CI)	P-value*
p.R853Q ($p = 8.63x10^{-3}$)	Hypsarrhythmia	HP:0002521	0.61	0.10	14.57 (4.83–47.16)	$3.75x10^{-7}$
	Epileptic spasms	HP:0011097	0.61	0.15	9.05 (3.06–28.75)	$1.64x10^{-5}$
	Infantile spasms	HP:0012469	0.56	0.14	7.51 (2.46–22.92)	$8.76x10^{-5}$
	Chorea	HP:0002072	0.22	0.01	27.10 (4.57–162.61)	$1.60x10^{-4}$
	Abnormality of the cerebral subcortex	HP:0010993	0.44	0.10	7.24 (2.34–21.77)	$2.86x10^{-4}$
	Hypoplasia of the corpus callosum	HP:0002079	0.28	0.03	11.13 (2.71–40.24)	$5.28x10^{-4}$
	Aplasia/Hypoplasia of the corpus callosum	HP:0007370	0.28	0.03	11.13 (2.71–40.24)	$5.28x10^{-4}$
	Vomiting	HP:0002013	0.17	0.01	37.91 (4.04–480.45)	$6.62x10^{-4}$
	Intellectual disability, severe	HP:0010864	0.61	0.23	5.30 (1.81–16.62)	$7.74x10^{-4}$
p.A263V ($p = 7.30x10^{-3}$)	Abnormality of eye movement	HP:0000496	0.36	0.02	30.08 (6.31–137.75)	$1.42x10^{-5}$
	Ataxia	HP0001251	0.50	0.06	14.72 (4.06–53.61)	$2.03x10^{-5}$
	Abnormality of coordination	HP:0011443	0.50	0.07	14.12 (3.91–51.27)	$2.53x10^{-5}$
	Inappropriate behavior	HP:0000719	0.21	0	Inf (12.98–Inf)	$3.12x10^{-5}$
	Inappropriate crying	HP:0030215	0.21	0	Inf (12.98–Inf)	$3.12x10^{-5}$
	Neonatal onset	HP:0003623	0.86	0.32	12.78 (2.78–119.30)	$6.94x10^{-5}$
	Abnormality of central motor function	HP:0011442	0.64	0.17	8.54 (2.48–33.49)	$1.80x10^{-4}$
	Abnormal eye physiology	HP:0012373	0.36	0.05	10.38 (2.50–38.54)	$7.47.x10^{-4}$
	Nystagmus	HP:0000639	0.21	0.01	26.06 (3.42–175.65)	0.001
	Abnormal involuntary eye movements	HP:0012547	0.21	0.01	26.06 (3.42–175.65)	0.001

p.L1342P ($p = 2.40 \times 10^{-4}$)	Poor eye contact	HP:0000817	0.60	0.02	56.63 (5.85–753.89)	2.36×10^{-4}
	Impaired social interactions	HP:0000735	0.60	0.03	51.59 (5.37–687.87)	3.00×10^{-4}
	Abnormal social behavior	HP:0012433	0.60	0.03	43.70 (4.61–557.11)	4.58×10^{-4}
	Brain very small	HP:0001322	0.80	0.10	34.20 (3.29–1701.95)	6.27×10^{-4}
	Atrophy/Degeneration affecting the CNS	HP:0007367	0.80	0.10	34.20 (3.29–1701.95)	6.27×10^{-4}
	Brain atrophy	HP:0012444	0.80	0.10	34.20 (3.29–1701.95)	6.27×10^{-4}
	Hypsarrhythmia	HP:0002521	0.80	0.11	31.70 (3.05–1576.99)	8.09×10^{-4}
p.M1545V ($p = 0.023$)	Generalized tonic-clonic seizures with focal onset	HP:0007334	0.50	0.01	91.80 (5.46–1664.05)	0.001
p.S1336Y ($p = 3.93 \times 10^{-3}$)	No significantly associated HPO terms					
p.E1211K ($p = 0.023$)	No significantly associated HPO terms					

*P-values for HPO terms associated with phenotypically significant recurrent variants identified 3 or more times in the cohort. All significantly associated terms that remained significant after correction for multiple testing.

Abbreviations: CI = confidence interval; CNS = central nervous system

Algorithm	*SCN2A* diagnosis groups	Missense versus PTV	Recurrent variants	*SCN2A* domains	*SCN2A* segments	Individuals reported with single publication	Average across categories
**Jaccard**	5.00	3.80	1.19	1.04	1.01	2.73	2.95
**Jaccard (weighted)**	2.27	2.66	1.24	0.83	0.86	2.71	2.11
**Chabalier**	2.19	2.41	1.23	0.79	0.76	2.63	2.00
**Wang (node)**	2.26	2.53	1.04	0.75	0.73	1.84	1.83
**Lin**	1.41	2.01	0.76	0.73	0.85	1.18	1.39
**Jiang and Conrath**	1.12	2.50	0.74	0.76	0.89	1.14	1.43
**Wang (edge)**	1.17	1.30	0.73	0.64	0.71	1.19	1.15
**Ganesan**	1.22	1.15	0.72	0.60	0.66	1.15	1.10
**Resnik**	1.23	1.23	0.69	0.61	0.62	1.10	1.10

Supplementary Fig. 8 Comparison of nine different phenotypic similarity algorithms across six different categories using the Global Similarity (GS) score, sorted by descending average GS score (green) across all six categories (blue). The Jaccard algorithm has a higher GS score in all categories while the conventional Resnik algorithm consistently yields the lowest GS score across all categories.

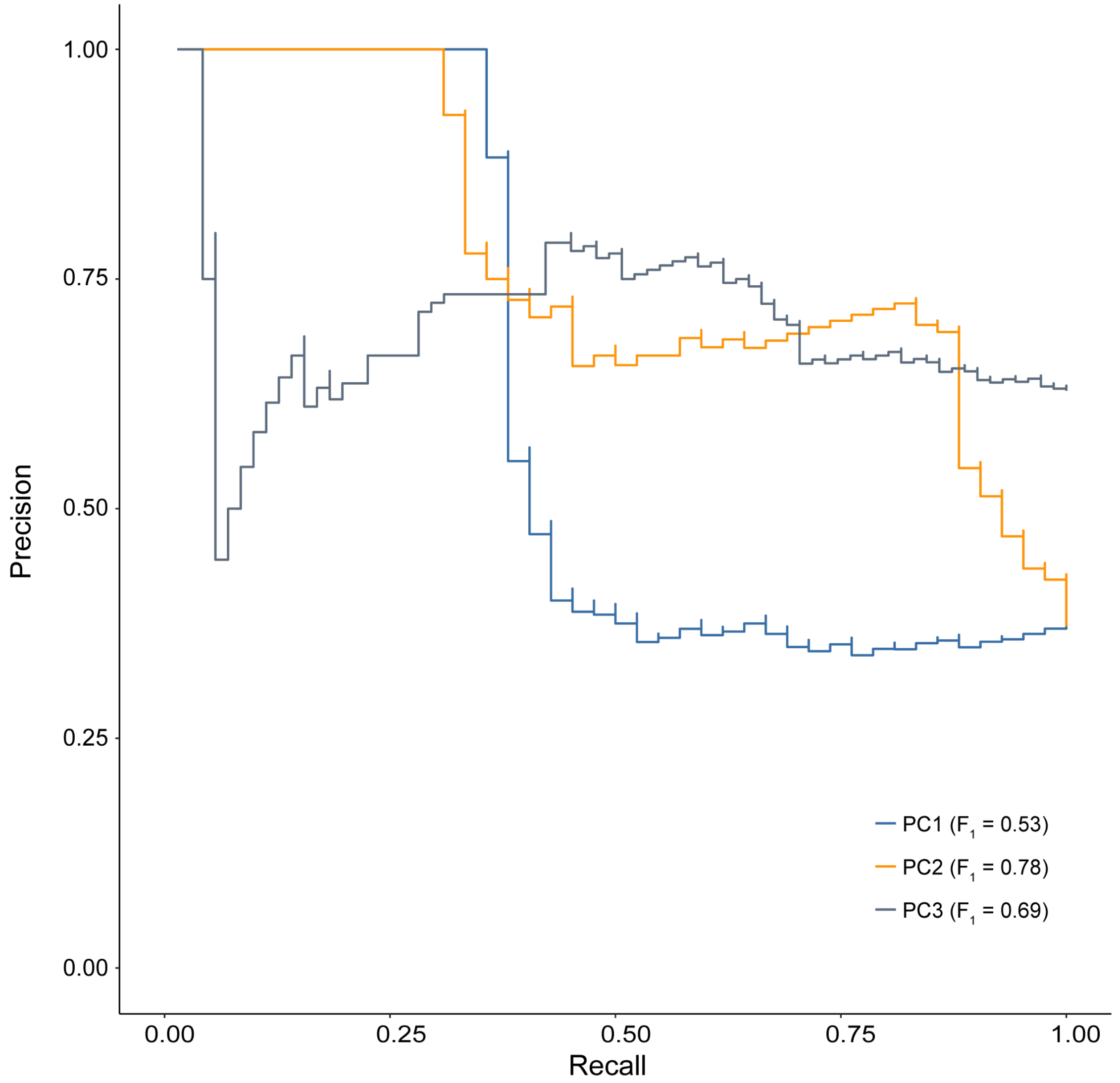

Supplementary Fig. 9 Precision-recall curves for the first three principal components of the computational phenotype analysis for the classification of gain-of-function versus loss-of-function variants. In addition to the ROC curve (Fig. 3), precision-recall curves were generated given the imbalance between the number of gain-of-function and loss-of-function variants. When jointly evaluating the precision-recall curve and F_1 scores, PC2 remained the principal component with the best performance to classify gain-of-function versus loss-of-function variants based on HPO-encoded phenotype data.

Supplementary Table 12. List of all variants included in study with references

Variant	Phenotypic and biochemical information sources
c.4551+1C>A	Current Study
A1006V	Current Study
A1316V	Parrini 2017[19]
A1333T	Olson 2017[20]
A1500T	Wolff 2017[10]
A1612G	Zeng 2018[21]
A1652P	Wolff 2017[10]
A1773T	Vidal 2019[7]
A1773V	Guo 2018[22], Wolff 2017[10]
A202V	Wolff 2017[10], Leroy 2018[23]
A240S	Wolff 2017[10], Howell 2015[24]
A263T	Wolff 2017[10], Nakamura 2013[25]
A263V	Schwarz 2019[5], Gorman 2017[26], Hader 2018[27], Parrini 2017[19], Wolff 2017[10], Nashabat 2019[28], Halvardson 2016[29], Liao 2010[30], Schwarz 2016[31], Johannesen 2016[32], Touma 2013[33]
A439fs*	Wolff 2017[10], Li 2015[34]
A467T	Liu 2018[35]
A733T	Wolff 2017[10]
A896V	Parrini 2017[19]
Arg275fs*	Kothur 2018[36]
c.2150-2A>G	Wolff 2017[10], Li 2015[34]
c.3521-2A>G	Kong 2019[37]
c.3850-2A>C	Wang 2016[8]
c.386+2T>C	Wolff 2017[10]
c.4551+1G	Wolff 2017[10], D'Gama 2016[38]
c.476+1G>A	Wolff 2017[10], Tavassoli 2014[39], Li 2015[34]
c.605+1G>A	Kothur 2018[36], Guo 2018[22], Wolff 2017[10]
c.698G>T	Wolff 2017[10]
C1170Vfs*15	Wolff 2017[10]
C1175Y	Kong 2019[37]
C1344Y	Parrini 2017[19]
C1386R	Ben-Shalom 2017[40], Iossifov 2014[41]
C959*	Wolff 2017[10], Li 2015[34], Sanders 2012[42]
D12N	Ben-Shalom 2017[40], Iossifov 2014[41]
D1487E	Guo 2018[22]
D1598G	Wolff 2017[10], Need 2012[43]
D195G	Current Study
D343G	Schwarz 2019[5], Wolff 2017[10]
D609*fs	Wolff 2017[10], Li 2015[34]
D649N	Wolff 2017[10], Wang 2012[8]
D82G	Ben-Shalom 2017[40], De Rubeis 2014[44]
D997Y	Kong 2019[37]

Del(exons_18-27)	Borlot 2019[45]
E1133D	Current Study
E1211K	Wolff 2017[10], Ogiwara 2009[46], Wong 2015[47], Grinton 2015[48], Kong 2019[37]
E1493del	Current Study
E169fs*	Wolff 2017[10], Carroll 2016[49]
E169G	Wolff 2017[10], Nakamura 2013[25]
E1803G	Papuc 2019[50]
E1880K	Ben-Shalom 2017[40], De Rubeis 2014[44]
E430A	Wolff 2017[10]
E430G	Olson 2017[20], Current Study
E430Q	Wolff 2017[10], Herlenius 2007[51]
E493del	Stosser 2018[52]
E717G*fs	Wolff 2017[10], Horvath 2016[53]
E999K	Wolff 2017[10], Allen 2015[54], Nakamura 2013[25], Trump 2016[55], Stosser 2018[52], Nashabat 2019[28], Miao 2018[56]
E999V	Wolff 2017[10], Trump 2016[55]
F1597L	Wolff 2017[10]
F1599Cfs*14	Wang 2016[8]
F1651C	Schwarz 2019[5], Wolff 2017[10]
F1682S	Kong 2019[37]
F1861Lfs*40	Vidal 2019[7]
F207S	Wolff 2017[10]
F328V	Ben-Shalom 2017[40], Saitoh 2015[57]
F612S	Wolff 2017[10]
F895S	Wolff 2017[10]
F928C	Wolff 2017[10], Howell 2015[24], Carvill 2013[58]
F978L	Ben-Shalom 2017[40], De Rubeis 2014[44]
G1013*	Wolff 2017[10], Li 2015[34], Sanders 2012[42]
G1223R	Wolff 2017[10]
G1460R	Yokoi 2018[59]
G1470A	Tammimies 2015[60]
G1522A	Wolff 2017[10], Mercimek-Malmutoglu 2015[61]
G1593R	Wolff 2017[10], Howell 2015[24]
G1634D	Ben-Shalom 2017[40], Leach 2016[62]
G1634V	Wolff 2017[10], Howell 2015[24]
G1744E	Wolff 2017[10]
G1744R	Wolff 2017[10], D'Gama 2016[38], Guo 2018[22]
G211D	Ben-Shalom 2017[40], Kodera 2013[63], Stosser 2018[52]
G478fs*	Ben-Shalom 2017[40], De Rubeis 2014[44]
G822S	Stosser 2018[52]
G828V	Wolff 2017[10]
G882E	Wolff 2017[10]
G882R	Wolff 2017[10]
G899S	Wolff 2017[10]
G999L	Foster 2017[64]

H1853R	Wolff 2017[10], Martin 2014[65]
H930Q	Wolff 2017[10]
I1021Yfs*	Wolff 2017[10], Carvill 2013[58], Howell 2015[24]
I1252V	Schwarz 2019[5]
I1281F	Wolff 2017[10]
I1334T; I1334N	Stosser 2018[52]
I1353M	Current Study
I1455N	Zhang 2017[66]
I1473M	Wolff 2017[10], Ogiwara 2009[46]
I1488N	Stosser 2018[52]
I1571T	Liu 2018[35], Wang 2019[67]
I1596S	Wolff 2017[10], Herlenius 2007[51]
I1615Rfs*47	Current Study
I1636M	Stosser 2018[52]
I1640F	Kong 2019[37]
I1640N	Current Study
I1640S	Wolff 2017[10]
I172V	Wolff 2017[10], Saitoh 2015[57]
I1772M	Guo 2018[22]
I237N	Wolff 2017[10]
I769T	Zhou 2017[68]
I873M	Wolff 2017[10], Trump 2016[55]
I874Mfs*5	Current Study
I890M	Kong 2019[37]
I891T	Wolff 2017[10]
K1260E; K1260Q	Wolff 2017[10], Trump 2016[55]
K1362M	Demos 2019[70]
K1387Sfs*4	Wolff 2017[10]
K1422E	Current Study
K1495E	Uzuner 2019[71]
K1641N	Wolff 2017[10], Zara 2013[72]
K1933M	Evers 2017[73], Wolff 2017[10]
K905N	Wolff 2017[10], Howell 2015[24], Carvill 2013[58]
K905Q	Butler 2017[74]
K908E	Wolff 2017[10], Lauxmann 2018[75]
L1003I	Wolff 2017[10], Berkovic 2004[76]
L1330F	Wolff 2017[10], Heron 2010[77]
L1341I	Current Study
L1341R	Ben-Shalom 2017[40]
L1342P	Wolff 2017[10], Matalon 2014[78], Hackenberg 2014[79], Dimassi 2015[80], Li 2015[34]
L1416F	Current Study
L1563V	Wolff 2017[10], Heron 2002[81]
L1614P	Current Study
L1650P	Wolff 2017[10], Trump 2016[55], Schwarz 2019[5], Fazeli 2018[82]
L1660W	Wolff 2017[10], Fukasawa 2015[83]

L1665F	Wolff 2017[10]
L1765P	Takezawa 2018[84]
L1829F	Wolff 2017[10], Trump 2016[55]
L216W	Ostrander 2018[85]
L248P	Chitre 2018[86]
L392L	Current Study
L421V	Cheng 2017[87]
L436S	Zeng 2018[21]
L501fs*	Wolff 2017[10], Li 2015[34]
L611Vfs*35	Wolff 2017[10], Rauch 2012[88]
L835F	Olson 2017[20]
L881P	de Kovel 2016[89], Wolff 2017[10]
L884H	Stosser 2018[52]
L983W	Ben-Shalom 2017[40], Pronicka 2016[90]
M1128T	Wolff 2017[10], Kobayashi 2012[91]
M1323V	Wolff 2017[10], Nakamura 2013[25], Parrini 2017[19]
M1338T	Wolff 2017[10] Nakamura 2013[25]
M136I	Turkdogan 2018[92], Wolff 2017[10], Howell 2015[24], Carvill 2013[58]
M1501V	Peng 2019[93]
M1545I	Hader 2018[27]
M1545V	Wolff 2017[10], Bruun 2017[94], Dilena 2017[95], Gokben 2017[96]
M1548T	Parrini 2017[19]
M1548V	Wolff 2017[10]
M1654I	Current Study
M1770L	Vlachuo 2019[97]
M1797Ifs*5	Butler 2017[74]
M1872I	Kong 2019[37]
M1879T	Zhang 2017[66]
M252V	Wolff 2017[10], Liao 2010[30]
N1001K	Wolff 2017[10], Striano 2006[98]
N132K	Wolff 2017[10], Matalon 2014[78]
N212D	Wolff 2017[10] Nakamura 2013[25]
N361Tfs*21	Guo 2018[22]
N503Kfs*19	Wolff 2017[10], Rauch 2012[88]
N876T	Wolff 2017[10], Nakamura 2013[25]
N976K	Wolff 2017[10], Howell 2015[24]
E438D	Current Study
E440Rfs*20	Wolff 2017[10], Jiang 2013[99]
F262S	Palmer 2018[100]
I1537_M1538delinsSI	Foster 2017[64]
P1622S	Wolff 2017[10]
P569A	Vidal 2019[7]
P70L	Kong 2019[37]
Q1479P	Wolff 2017[10], Trump 2016[55]
Q1521fs*	Wolff 2017[10], Li 2015[34], Ben-Shalom 2017[40], Gilissen 2014[101], de Ligt 2012[102]

Q1531K	Wolff 2017[10], Grinton 2015[48]
Q169G	Iwama 2019[103]
Q1803G	Papuc 2019[50]
Q1811E	Wolff 2017[10]
Q1904Rfs*22	Wang 2016[8]
Q383E	Syrbe 2016[104]
Q430G	Wolff 2017[10], Matalon 2014[78]
Q901E	Stosser 2018[52]
R102fs*	Ben-Shalom 2017[40], De Rubeis 2014[44], Monies 2017[105], Wolff 2017[10], Kamiya 2004[106]
R1235*	Wolff 2017[10]
R1312T	Wolff 2017[10], Shi 2009[107]
R1319L	Nashabat 2019[28], Zhang 2017[66]
R1319P	Wang 2016[8]
R1319Q	Wolff 2017[10], Berkovic 2004[76]
R1319W	Moller 2016[108], Wolff 2017[10]
R1435fs*	Wolff 2017[10], Trump 2016[55], Guo 2018[22]
R1515fs*	Wolff 2017[10], D'Gama 2016[38]
R1626Q	Willig 2015[109]
R1629H	Kong 2019[37], Wolff 2017[10], Nashabat 2019[28]
R1629L	Wolff 2017[10], Nakamura 2013[25]
R1632K	Stosser 2018[52]
R1635Q	Guo 2018[22]
R1882G	Wolff 2017[10], Schwarz 2016[31]
R1882L	Wolff 2017[10], Baasch 2014[110]
R1882P	Wolff 2017[10]
R1882Q	Wolff 2017[10], Howell 2015[24], Trump 2016[55], Carvill 2013[58], Berecki 2018[111], Parrini 2017[19], Butler 2017[74]
R188W	Ito 2006[112], Wolff 2017[10], Sugawara 2001[113]
R1902C	Wolff 2017[10], Weiss 2003[114]
R1918H	Wolff 2017[10], Haug 2001[115]
R220G	Wolff 2017[10], Mercimek-Malmutoglu 2015[61]
R223Q	Zeng 2018[21], Wolff 2017[10], Berkovic 2004[76]
R28C	Wolff 2017[10], Jiang 2013[99]
R36G	Kong 2019[37], Wolff 2017[10]
R379H	Ben-Shalom 2017[40], De Rubeis 2014[44]
R395P	Guo 2018[22]
R524*	Current Study
R583*	Wolff 2017[10], Codina-Sola 2015[116]
R850P	Ben-Shalom 2017[40], Carroll 2016[49]
R853Q	Wolff 2017[10], Samanta 2015, Nakamura 2013[25], Li 2015[34], Epi4K 2013[117], Kobayashi 2016[118], Ben-Shalom 2017[40], Kong 2019[37], Berecki 2018[111], Cherot 2018[119], Butler 2017[74], Vidal 2019[7]
R856*	Diets 2018, Guo 2018[22]
R856L	Wolff 2017[10], Howell 2015[24]
R856Q	Wolff 2017[10], Buers 2019[120], Moller 2016[108]

R922C	Guo 2018[22]
R937C	Wolff 2017[10], Rauch 2012[88], Li 2015[34], Iossifov 2014[41], De Rubeis 2014[44], Ben-Shalom 2017[40]
R937H	Guo 2018[22], Ben-Shalom 2017[40]
S1318P	Xiao 2017[121]
S1336Y	Wolff 2017[10], Zerem 2014[122], Nakamura 2013[25]
S1536R	Wolff 2017[10]
S1584C	Kong 2019[37]
S1656F	Wolff 2017[10]
S1656P	Suddaby 2019[123]
S1758R	Guo 2018[22], Current Study
S214P	Moller 2016[108]
S488fs*	Yamamoto 2019[124]
S565fs*	Ben-Shalom 2017[40], De Rubeis 2014[44]
S579R	Zeng 2018[21]
S686fs*	Ben-Shalom 2017[40], De Rubeis 2014[44]
S863F	Wolff 2017[10]
S987I	Wolff 2017[10], Trump 2016[55], Schwarz 2019[5], Fokstuen 2016[125]
T1420M	Wolff 2017[10], Li 2015[34], Ben-Shalom 2017[40], Iossifov 2014[41]
T1464A	Nashabat 2019[28]
T1623N	Wolff 2017[10], Nakamura 2013[25], Li 2015[34]
T1711Lfs*8	Wolff 2017[10]
T185I	Current Study
T218K	Wolff 2017[10], Howell 2015[24]
T227I	Palmer 2018[100], Wolff 2017[10]
T236S	Wolff 2017[10], Nakamura 2013[25]
T365M	Guo 2018[22]
T393K	Butler 2017[74]
T400R	Current Study
T435Ifs*5	Guo 2018[22]
T674K	Wolff 2017[10], D'Gama 2016[38]
T773I	Lauxmann 2018[75]
T784Cfs*45	Lee 2018[126]
V1261M	Moller 2016[108]
V1263M	Moller 2016[108]
V1282F	Wolff 2017[10], Carroll 2016[49]
V1325F	Schwarz 2019[5], Maksemous 2018[127]
V1325I	Schwarz 2019[5]
V1326D	Wolff 2017[10], Dhamija 2013[128]
V1326L	Wolff 2017[10], Nakamura 2013[25]
V1528Cfs*7	Moller 2016[108], Wolff 2017[10]
V1607A	Huang 2019[129]
V1627M	Wolff 2017[10]
V208E	Lauxmann 2018[75], Wolff 2017[10], Lemke 2012[130]
V213A	Nashabat 2019[28]

V213D	Wolff 2017[10], Nakamura 2013[25]
V251A	Zeng 2018[21]
V251I	Bernardo 2017[131], Parrini 2017[19]
V261M	Wolff 2017[10], Liao 2010[30], Zhang 2017[66], Kong 2019[37]
V423L	Wolff 2017[10]
V424L	Wolff 2017[10]
V424M	Liang 2017[132]
V887A	Wolff 2017[10]
V892I	Wolff 2017[10], Berkovic 2004[76]
W1276fs*	Trujillano 2017[133]
W1348fs*	Vidal 2019[7]
W1398fs*	Wolff 2017[10], Li 2015[34], Ben-Shalom 2017[40], de Ligt 2012[102]
W1594R	Ben-Shalom 2017[40], De Rubeis 2014[44]
W1716fs*	Wolff 2017[10]
W191C	Su 2018[134]
W191G	Parrini 2017[19]
W281fs*	Wolff 2017[10]
Y1589C	Wolff 2017[10], Lauxmann 2013[135]
Y1771H	Schwarz 2019[5]
Y428C	Sahli 2019[136]

Supplementary References

1. Andrews T, Meader S, Vulto-van Silfhout A, et al. Gene networks underlying convergent and pleiotropic phenotypes in a large and systematically-phenotyped cohort with heterogeneous developmental disorders. PLoS Genet. 2015;11(3):e1005012.
2. Helbig I, Lopez-Hernandez T, Shor O, et al. A Recurrent Missense Variant in AP2M1 Impairs Clathrin-Mediated Endocytosis and Causes Developmental and Epileptic Encephalopathy. Am J Hum Genet. 2019;104(6):1060-1072.
3. Galer P, Ganesan S, Lewis-Smith D, et al. Semantic similarity analysis reveals robust gene-disease relationships in developmental and epileptic encephalopathies. Am J Hum Genet. 2020.
4. Papuc SM, Abela L, Steindl K, et al. The role of recessive inheritance in early-onset epileptic encephalopathies: a combined whole-exome sequencing and copy number study. Eur J Hum Genet. 2019;27(3):408-421.
5. Schwarz N, Bast T, Gaily E, et al. Clinical and genetic spectrum of SCN2A-associated episodic ataxia. Eur J Paediatr Neurol. 2019;23(3):438-447.
6. Berecki G, Howell KB, Deerasooriya YH, et al. Dynamic action potential clamp predicts functional separation in mild familial and severe de novo forms of SCN2A epilepsy. Proc Natl Acad Sci U S A. 2018;115(24):E5516-E5525.
7. Vidal S, Brandi N, Pacheco P, et al. The most recurrent monogenic disorders that overlap with the phenotype of Rett syndrome. Eur J Paediatr Neurol. 2019;23(4):609-620.
8. Wang TY, Guo H, Xiong B, et al. De novo genic mutations among a Chinese autism spectrum disorder cohort. Nat Commun. 2016;7:10 (Article).
9. Kong Y, Yan K, Hu L, et al. Data on mutations and Clinical features in SCN1A or SCN2A gene. Data Brief. 2019;22:492-501.
10. Wolff M, Johannesen KM, Hedrich UBS, et al. Genetic and phenotypic heterogeneity suggest therapeutic implications in SCN2A-related disorders. Brain. 2017;140(5):1316-1336.
11. Pesquita C, Faria D, Falcao AO, Lord P, Couto FM. Semantic similarity in biomedical ontologies. PLoS Comput Biol. 2009;5(7):e1000443.
12. Lin D. An information-theoretic definition of similarity. Paper presented at: 15th International Conference on Machine Learning1998; San Francisco.
13. Jiang J, Conrath D. Semantic similarity based on corpus statistics and lexical taxonomy. Paper presented at: 10th International Conference on Research on Computational Linguistics1997; Taiwan.
14. Wang JZ, Du Z, Payattakool R, Yu PS, Chen CF. A new method to measure the semantic similarity of GO terms. Bioinformatics. 2007;23(10):1274-1281.
15. Chabalier J, Mosser J, Burgun A. A transversal approach to predict gene product networks from ontology-based similarity. BMC Bioinformatics. 2007;8:235.
16. Ganesan S, Galer PD, Helbig KL, et al. A longitudinal footprint of genetic epilepsies using automated electronic medical record interpretation. Genet Med. 2020;22(12):2060-2070.
17. Saito T, Rehmsmeier M. The precision-recall plot is more informative than the ROC plot when evaluating binary classifiers on imbalanced datasets. PLoS One. 2015;10(3):e0118432.
18. Sing T, Sander O, Beerenwinkel N, Lengauer T. ROCR: visualizing classifier performance in R. Bioinformatics. 2005;21(20):3940-3941.
19. Parrini E, Marini C, Mei D, et al. Diagnostic Targeted Resequencing in 349 Patients with Drug-Resistant Pediatric Epilepsies Identifies Causative Mutations in 30 Different Genes. Hum Mutat. 2017;38(2):216-225.
20. Olson HE, Kelly M, LaCoursiere CM, et al. Genetics and Genotype-Phenotype Correlations in Early Onset Epileptic Encephalopathy with Burst Suppression. Ann Neurol. 2017;81(3):419-429 (Article).
21. Zeng Q, Yang XL, Zhang J, et al. Genetic analysis of benign familial epilepsies in the first year of life in a Chinese cohort. J Hum Genet. 2018;63(1):9-18 (Article).
22. Guo H, Wang T, Wu H, et al. Inherited and multiple de novo mutations in autism/developmental delay risk genes suggest a multifactorial model. Mol Autism. 2018;9:64-64.

23. Leroy A, Corfiotti C, Nguyen The Tich S, et al. Catatonia Associated With a SCN2A-Related Disorder in a 4-Year-Old Child. Pediatrics. 2018;142(5):e20181231.
24. Howell K, McMahon J, Carvill G. SCN2A encephalopathy. A major cause of epilepsy in infancy with migrating focal seizures. Neurology 2015;85:958-966. Pediatr Neurol. 2016;59:4-4 (Editorial Material).
25. Nakamura K, Kato M, Osaka H, et al. Clinical spectrum of SCN2A mutations expanding to Ohtahara syndrome. Neurology. 2013;81(11):992-998 (Article).
26. Gorman KM, King MD. SCN2A p.Ala263Val Variant a Phenotype of Neonatal Seizures Followed by Paroxysmal Ataxia in Toddlers. Pediatr Neurol. 2017;67:111-112.
27. Flor-Hirsch H, Heyman E, Livneh A, et al. Lacosamide for SCN2A-related intractable neonatal and infantile seizures. Epileptic disorders : international epilepsy journal with videotape. 2018;20(5):440-446.
28. Nashabat M, Al Qahtani XS, Almakdob S, et al. The landscape of early infantile epileptic encephalopathy in a consanguineous population. Seizure. 2019;69:154-172.
29. Halvardson J, Zhao JJ, Zaghlool A, et al. Mutations in HECW2 are associated with intellectual disability and epilepsy. J Med Genet. 2016;53(10):697-704.
30. Liao Y, Anttonen AK, Liukkonen E, et al. SCN2A mutation associated with neonatal epilepsy, late-onset episodic ataxia, myoclonus, and pain. Neurology. 2010;75(16):1454-1458 (Article).
31. Schwarz N, Hahn A, Bast T, et al. Mutations in the sodium channel gene SCN2A cause neonatal epilepsy with late-onset episodic ataxia. Journal of neurology. 2016;263(2):334-343.
32. Johannesen KM, Miranda MJ, Lerche H, Møller RS. Letter to the editor: confirming neonatal seizure and late onset ataxia in SCN2A Ala263Val. J Neurol. 2016;263(7):1459-1460.
33. Touma M, Joshi M, Connolly MC, et al. Whole genome sequencing identifies SCN2A mutation in monozygotic twins with Ohtahara syndrome and unique neuropathologic findings. Epilepsia. 2013;54(5):e81-85.
34. Li JC, Cai T, Jiang Y, et al. Genes with de novo mutations are shared by four neuropsychiatric disorders discovered from NPdenovo database. Mol Psychiatr. 2016;21(2):290-297 (Article).
35. Liu N, Schoch K, Luo X, et al. Functional variants in TBX2 are associated with a syndromic cardiovascular and skeletal developmental disorder. Human Molecular Genetics. 2018;27(14):2454-2465.
36. Kothur K, Holman K, Farnsworth E, et al. Diagnostic yield of targeted massively parallel sequencing in children with epileptic encephalopathy. Seizure. 2018;59:132-140.
37. Kong Y, Yan K, Hu L, et al. Data on mutations and Clinical features in SCN1A or SCN2A gene. Data in Brief. 2019;22:492-501.
38. D'Gama AM, Geng Y, Couto JA, et al. Mammalian Target of Rapamycin Pathway Mutations Cause Hemimegalencephaly and Focal Cortical Dysplasia. Ann Neurol. 2015;77(4):720-725.
39. Tavassoli T, Kolevzon A, Wang AT, et al. De novo SCN2A splice site mutation in a boy with Autism spectrum disorder. BMC medical genetics. 2014;15:35.
40. Ben-Shalom R, Keeshen CM, Berrios KN, An JY, Sanders SJ, Bender KJ. Opposing Effects on Na(V)1.2 Function Underlie Differences Between SCN2A Variants Observed in Individuals With Autism Spectrum Disorder or Infantile Seizures. Biol Psychiatry. 2017;82(3):224-232 (Article).
41. Iossifov I, O'Roak BJ, Sanders SJ, et al. The contribution of de novo coding mutations to autism spectrum disorder. Nature. 2014;515(7526):216-221.
42. Sanders SJ, Murtha MT, Gupta AR, et al. De novo mutations revealed by whole-exome sequencing are strongly associated with autism. Nature. 2012;485(7397):237-241.
43. Need AC, Shashi V, Hitomi Y, et al. Clinical application of exome sequencing in undiagnosed genetic conditions. J Med Genet. 2012;49(6):353-361.
44. De Rubeis S, He X, Goldberg AP, et al. Synaptic, transcriptional and chromatin genes disrupted in autism. Nature. 2014;515(7526):209-215.
45. Borlot F, de Almeida BI, Combe SL, Andrade DM, Filloux FM, Myers KA. Clinical utility of multigene panel testing in adults with epilepsy and intellectual disability. Epilepsia. 2019;60(8):1661-1669.
46. Ogiwara I, Ito K, Sawaishi Y, et al. De novo mutations of voltage-gated sodium channel alphaII gene SCN2A in intractable epilepsies. Neurology. 2009;73(13):1046-1053.
47. Wong VC, Fung CW, Kwong AK. SCN2A mutation in a Chinese boy with infantile spasm - response to Modified Atkins Diet. Brain & development. 2015;37(7):729-732.

48. Grinton BE, Heron SE, Pelekanos JT, et al. Familial neonatal seizures in 36 families: Clinical and genetic features correlate with outcome. Epilepsia. 2015;56(7):1071-1080.
49. Carroll LS, Woolf R, Ibrahim Y, et al. Mutation screening of SCN2A in schizophrenia and identification of a novel loss-of-function mutation. Psychiatr Genet. 2016;26(2):60-65 (Article).
50. Papuc SM, Abela L, Steindl K, et al. The role of recessive inheritance in early-onset epileptic encephalopathies: a combined whole-exome sequencing and copy number study. Eur J Hum Genet. 2019;27(3):408-421.
51. Herlenius E, Heron SE, Grinton BE, et al. SCN2A mutations and benign familial neonatal-infantile seizures: the phenotypic spectrum. Epilepsia. 2007;48(6):1138-1142.
52. Stosser MB, Lindy AS, Butler E, et al. High frequency of mosaic pathogenic variants in genes causing epilepsy-related neurodevelopmental disorders. Genet Med. 2018;20(4):403-410 (Article).
53. Horvath GA, Demos M, Shyr C, et al. Secondary neurotransmitter deficiencies in epilepsy caused by voltage-gated sodium channelopathies: A potential treatment target? Molecular genetics and metabolism. 2016;117(1):42-48.
54. Allen NM, Conroy J, Shahwan A, et al. Unexplained early onset epileptic encephalopathy: Exome screening and phenotype expansion. Epilepsia. 2016;57(1):E12-E17 (Article).
55. Trump N, McTague A, Brittain H, et al. Improving diagnosis and broadening the phenotypes in early-onset seizure and severe developmental delay disorders through gene panel analysis. J Med Genet. 2016;53(5):310-317 (Article).
56. Miao P, Feng J, Guo Y, et al. Genotype and phenotype analysis using an epilepsy-associated gene panel in Chinese pediatric epilepsy patients. Clinical genetics. 2018;94(6):512-520.
57. Saitoh M, Shinohara M, Ishii A, et al. Clinical and genetic features of acute encephalopathy in children taking theophylline. Brain and Development. 2015;37(5):463-470.
58. Carvill GL, Heavin SB, Yendle SC, et al. Targeted resequencing in epileptic encephalopathies identifies de novo mutations in CHD2 and SYNGAP1. Nature genetics. 2013;45(7):825-830.
59. Yokoi T, Enomoto Y, Tsurusaki Y, Naruto T, Kurosawa K. Nonsyndromic intellectual disability with novel heterozygous SCN2A mutation and epilepsy. Hum Genome Var. 2018;5:20-20.
60. Tammimies K, Marshall CR, Walker S, et al. Molecular Diagnostic Yield of Chromosomal Microarray Analysis and Whole-Exome Sequencing in Children With Autism Spectrum Disorder. Jama. 2015;314(9):895-903.
61. Mercimek-Mahmutoglu S, Patel J, Cordeiro D, et al. Diagnostic yield of genetic testing in epileptic encephalopathy in childhood. Epilepsia. 2015;56(5):707-716.
62. Leach EL, van Karnebeek CDM, Townsend KN, Tarailo-Graovac M, Hukin J, Gibson WT. Episodic ataxia associated with a de novo SCN2A mutation. European Journal of Paediatric Neurology. 2016;20(5):772-776.
63. Kodera H, Kato M, Nord AS, et al. Targeted capture and sequencing for detection of mutations causing early onset epileptic encephalopathy. Epilepsia. 2013;54(7):1262-1269.
64. Foster LA, Johnson MR, MacDonald JT, et al. Infantile Epileptic Encephalopathy Associated With SCN2A Mutation Responsive to Oral Mexiletine. Pediatr Neurol. 2017;66:108-111.
65. Martin HC, Kim GE, Pagnamenta AT, et al. Clinical whole-genome sequencing in severe early-onset epilepsy reveals new genes and improves molecular diagnosis. Human molecular genetics. 2014;23(12):3200-3211.
66. Zhang Q, Li J, Zhao Y, Bao X, Wei L, Wang J. Gene mutation analysis of 175 Chinese patients with early-onset epileptic encephalopathy. Clinical genetics. 2017;91(5):717-724.
67. Wang J, Wen Y, Zhang Q, et al. Gene mutational analysis in a cohort of Chinese children with unexplained epilepsy: Identification of a new KCND3 phenotype and novel genes causing Dravet syndrome. Seizure. 2019;66:26-30.
68. Zhou P, He N, Zhang JW, et al. Novel mutations and phenotypes of epilepsy-associated genes in epileptic encephalopathies. Genes, brain, and behavior. 2018;17(8):e12456.
69. Wolff M, Johannesen KM, Hedrich UBS, et al. GENETIC AND PHENOTYPIC HETEROGENEITY SUGGEST THERAPEUTIC IMPLICATIONS IN SCN2A-RELATED DISORDERS. Epilepsia. 2017;58:S12-S13 (Meeting Abstract).

70. Demos M, Guella I, DeGuzman C, et al. Diagnostic Yield and Treatment Impact of Targeted Exome Sequencing in Early-Onset Epilepsy. Front Neurol. 2019;10:434-434.
71. Uzuner G. Erken başlangıçlı Epileptik Ensefalopati hastalarında hedefli gen paneli analizlerinin tanısal değerinin saptanması. Kocaeli Universitesi, Saglik Bilimleri Enstitusu; 2019.
72. Zara F, Specchio N, Striano P, et al. Genetic testing in benign familial epilepsies of the first year of life: clinical and diagnostic significance. Epilepsia. 2013;54(3):425-436.
73. Evers C, Staufner C, Granzow M, et al. Impact of clinical exomes in neurodevelopmental and neurometabolic disorders. Molecular genetics and metabolism. 2017;121(4):297-307.
74. Butler KM, da Silva C, Alexander JJ, Hegde M, Escayg A. Diagnostic Yield From 339 Epilepsy Patients Screened on a Clinical Gene Panel. Pediatr Neurol. 2017;77:61-66.
75. Lauxmann S, Verbeek NE, Liu Y, et al. Relationship of electrophysiological dysfunction and clinical severity in SCN2A-related epilepsies. Hum Mutat. 2018;39(12):1942-1956.
76. Berkovic SF, Heron SE, Giordano L, et al. Benign familial neonatal-infantile seizures: characterization of a new sodium channelopathy. Ann Neurol. 2004;55(4):550-557.
77. Heron SE, Scheffer IE, Grinton BE, et al. Familial neonatal seizures with intellectual disability caused by a microduplication of chromosome 2q24.3. Epilepsia. 2010;51(9):1865-1869.
78. Matalon D, Goldberg E, Medne L, Marsh ED. Confirming an expanded spectrum of SCN2A mutations: a case series. Epileptic disorders : international epilepsy journal with videotape. 2014;16(1):13-18.
79. Hackenberg A, Baumer A, Sticht H, et al. Infantile Epileptic Encephalopathy, Transient Choreoathetotic Movements, and Hypersomnia due to a De Novo Missense Mutation in the SCN2A Gene. Vol 452014.
80. Dimassi S, Labalme A, Ville D, et al. Whole-exome sequencing improves the diagnosis yield in sporadic infantile spasm syndrome. Clinical genetics. 2016;89(2):198-204.
81. Heron SE, Crossland KM, Andermann E, et al. Sodium-channel defects in benign familial neonatal-infantile seizures. Lancet. 2002;360(9336):851-852.
82. Fazeli W, Becker K, Herkenrath P, et al. Dominant SCN2A Mutation Causes Familial Episodic Ataxia and Impairment of Speech Development. Neuropediatrics. 2018;49(6):379-384 (Article).
83. Fukasawa T, Kubota T, Negoro T, et al. A case of recurrent encephalopathy with SCN2A missense mutation. Vol 372014.
84. Takezawa Y, Kikuchi A, Haginoya K, et al. Genomic analysis identifies masqueraders of full-term cerebral palsy. Ann Clin Transl Neurol. 2018;5(5):538-551.
85. Ostrander BEP, Butterfield RJ, Pedersen BS, et al. Whole-genome analysis for effective clinical diagnosis and gene discovery in early infantile epileptic encephalopathy. NPJ Genom Med. 2018;3:22-22.
86. Chitre M, Nahorski MS, Stouffer K, et al. PEHO syndrome: the endpoint of different genetic epilepsies. J Med Genet. 2018;55(12):803-813.
87. Cheng Y, Zhang L, Huang X, et al. De novo SCN2A mutation in a Chinese infant with severe early-onset epileptic encephalopathy, bronchopulmonary dysplasia, and adrenal hypofunction. Int J Clin Exp Pathol. 2017;10(10):10358-10362.
88. Rauch A, Wieczorek D, Graf E, et al. Range of genetic mutations associated with severe non-syndromic sporadic intellectual disability: an exome sequencing study. The Lancet. 2012;380(9854):1674-1682.
89. de Kovel CGF, Brilstra EH, van Kempen MJA, et al. Targeted sequencing of 351 candidate genes for epileptic encephalopathy in a large cohort of patients. Mol Genet Genomic Med. 2016;4(5):568-580.
90. Pronicka E, Piekutowska-Abramczuk D, Ciara E, et al. New perspective in diagnostics of mitochondrial disorders: two years' experience with whole-exome sequencing at a national paediatric centre. Journal of translational medicine. 2016;14(1):174.
91. Kobayashi K, Ohzono H, Shinohara M, et al. Acute encephalopathy with a novel point mutation in the SCN2A gene. Epilepsy Research. 2012;102(1):109-112.
92. Turkdogan D, Thomas G, Demirel B. Ketogenic diet as a successful early treatment modality for SCN2A mutation. Brain & development. 2019;41(4):389-391.
93. Peng J, Pang N, Wang Y, et al. Next-generation sequencing improves treatment efficacy and reduces hospitalization in children with drug-resistant epilepsy. CNS Neurosci Ther. 2019;25(1):14-20.
94. Bruun TUJ, DesRoches CL, Wilson D, et al. Prospective cohort study for identification of underlying genetic causes in neonatal encephalopathy using whole-exome sequencing. Genet Med. 2018;20(5):486-494.

95. Dilena R, Striano P, Gennaro E, et al. Efficacy of sodium channel blockers in SCN2A early infantile epileptic encephalopathy. Brain & development. 2017;39(4):345-348.
96. Gokben S, Onay H, Yilmaz S, et al. Targeted next generation sequencing: the diagnostic value in early-onset epileptic encephalopathy. Acta neurologica Belgica. 2017;117(1):131-138.
97. Vlachou V, Larsen L, Pavlidou E, et al. SCN2A mutation in an infant with Ohtahara syndrome and neuroimaging findings: expanding the phenotype of neuronal migration disorders. Journal of genetics. 2019;98(2).
98. Striano P, Bordo L, Lispi ML, et al. A novel SCN2A mutation in family with benign familial infantile seizures. Epilepsia. 2006;47(1):218-220.
99. Jiang Y-h, Yuen RKC, Jin X, et al. Detection of clinically relevant genetic variants in autism spectrum disorder by whole-genome sequencing. American journal of human genetics. 2013;93(2):249-263.
100. Palmer EE, Schofield D, Shrestha R, et al. Integrating exome sequencing into a diagnostic pathway for epileptic encephalopathy: Evidence of clinical utility and cost effectiveness. Mol Genet Genomic Med. 2018;6(2):186-199.
101. Gilissen C, Hehir-Kwa JY, Thung DT, et al. Genome sequencing identifies major causes of severe intellectual disability. Nature. 2014;511(7509):344-347.
102. de Ligt J, Willemsen MH, van Bon BW, et al. Diagnostic exome sequencing in persons with severe intellectual disability. The New England journal of medicine. 2012;367(20):1921-1929.
103. Iwama K, Mizuguchi T, Takeshita E, et al. Genetic landscape of Rett syndrome-like phenotypes revealed by whole exome sequencing. J Med Genet. 2019;56(6):396-407.
104. Syrbe S, Zhorov BS, Bertsche A, et al. Phenotypic Variability from Benign Infantile Epilepsy to Ohtahara Syndrome Associated with a Novel Mutation in SCN2A. Mol Syndromol. 2016;7(4):182-188.
105. Monies D, Abouelhoda M, AlSayed M, et al. The landscape of genetic diseases in Saudi Arabia based on the first 1000 diagnostic panels and exomes. Hum Genet. 2017;136(8):921-939.
106. Kamiya K, Kaneda M, Sugawara T, et al. A nonsense mutation of the sodium channel gene SCN2A in a patient with intractable epilepsy and mental decline. J Neurosci. 2004;24(11):2690-2698.
107. Shi X, Yasumoto S, Nakagawa E, Fukasawa T, Uchiya S, Hirose S. Missense mutation of the sodium channel gene SCN2A causes Dravet syndrome. Brain & development. 2009;31(10):758-762.
108. Møller RS, Larsen LH, Johannesen KM, et al. Gene Panel Testing in Epileptic Encephalopathies and Familial Epilepsies. Mol Syndromol. 2016;7(4):210-219.
109. Willig LK, Petrikin JE, Smith LD, et al. Whole-genome sequencing for identification of Mendelian disorders in critically ill infants: a retrospective analysis of diagnostic and clinical findings. Lancet Respir Med. 2015;3(5):377-387.
110. Baasch AL, Hüning I, Gilissen C, et al. Exome sequencing identifies a de novo SCN2A mutation in a patient with intractable seizures, severe intellectual disability, optic atrophy, muscular hypotonia, and brain abnormalities. Epilepsia. 2014;55(4):e25-29.
111. Berecki G, Howell KB, Deerasooriya YH, et al. Dynamic action potential clamp predicts functional separation in mild familial and severe de novo forms of SCN2A epilepsy. Proc Natl Acad Sci U S A. 2018;115(24):E5516-E5525 (Article).
112. Ito M, Yamakawa K, Sugawara T, Hirose S, Fukuma G, Kaneko S. Phenotypes and genotypes in epilepsy with febrile seizures plus. Epilepsy Research. 2006;70:199-205.
113. Sugawara T, Tsurubuchi Y, Agarwala KL, et al. A missense mutation of the Na+ channel alpha II subunit gene Na(v)1.2 in a patient with febrile and afebrile seizures causes channel dysfunction. Proc Natl Acad Sci U S A. 2001;98(11):6384-6389.
114. Weiss LA, Escayg A, Kearney JA, et al. Sodium channels SCN1A, SCN2A and SCN3A in familial autism. Mol Psychiatry. 2003;8(2):186-194.
115. Haug K, Hallmann K, Rebstock J, et al. The voltage-gated sodium channel gene SCN2A and idiopathic generalized epilepsy. Epilepsy Res. 2001;47(3):243-246.
116. Codina-Sola M, Rodriguez-Santiago B, Homs A, et al. Integrated analysis of whole-exome sequencing and transcriptome profiling in males with autism spectrum disorders. Mol Autism. 2015;6:16 (Article).
117. Epi KC, Epilepsy Phenome/Genome P, Allen AS, et al. De novo mutations in epileptic encephalopathies. Nature. 2013;501(7466):217-221.

118. Kobayashi Y, Tohyama J, Kato M, et al. High prevalence of genetic alterations in early-onset epileptic encephalopathies associated with infantile movement disorders. Vol 382015.
119. Chérot E, Keren B, Dubourg C, et al. Using medical exome sequencing to identify the causes of neurodevelopmental disorders: Experience of 2 clinical units and 216 patients. Clinical genetics. 2018;93(3):567-576.
120. Buers I, Persico I, Schöning L, et al. Crisponi/cold-induced sweating syndrome: Differential diagnosis, pathogenesis and treatment concepts. Clinical genetics. 2020;97(1):209-221.
121. Xiao B, Qiu W, Ji X, et al. Marked yield of re-evaluating phenotype and exome/target sequencing data in 33 individuals with intellectual disabilities. American journal of medical genetics Part A. 2018;176(1):107-115.
122. Zerem A, Lev D, Blumkin L, et al. Paternal germline mosaicism of a SCN2A mutation results in Ohtahara syndrome in half siblings. Eur J Paediatr Neurol. 2014;18(5):567-571.
123. Suddaby JS, Silver J, So J. Understanding the schizophrenia phenotype in the first patient with the full SCN2A phenotypic spectrum. Psychiatr Genet. 2019.
124. Yamamoto T, Imaizumi T, Yamamoto-Shimojima K, et al. Genomic backgrounds of Japanese patients with undiagnosed neurodevelopmental disorders. Brain & development. 2019;41(9):776-782.
125. Fokstuen S, Makrythanasis P, Hammar E, et al. Experience of a multidisciplinary task force with exome sequencing for Mendelian disorders. Hum Genomics. 2016;10(1):24-24.
126. Lee H-CH, Lau NK-C, Yeung C-W, Ng S-FG, Yau K-CE, Mak CM. Successful Adaptation of Targeted Gene Panel Next-Generation Sequencing in Regional Hospital in Hong Kong: Genomic Diagnosis of SCN2A-Related Seizure Disorder. Chin Med J (Engl). 2018;131(18):2262-2264.
127. Maksemous N, Smith RA, Sutherland HG, Sampaio H, Griffiths LR. Whole-Exome Sequencing Implicates SCN2A in Episodic Ataxia, but Multiple Ion Channel Variants May Contribute to Phenotypic Complexity. Int J Mol Sci. 2018;19(10):3113.
128. Dhamija R, Wirrell E, Falcao G, Kirmani S, Wong-Kisiel LC. Novel de novo SCN2A Mutation in a Child With Migrating Focal Seizures of Infancy. Pediatr Neurol. 2013;49(6):486-488.
129. Huang Q, Yu L, Ma M, Qi H, Wu Y. Novel SCN2A mutation in a family associated with juvenile-onset myoclonus: Case report. Medicine (Baltimore). 2019;98(8):e14698-e14698.
130. Lemke JR, Riesch E, Scheurenbrand T, et al. Targeted next generation sequencing as a diagnostic tool in epileptic disorders. Epilepsia. 2012;53(8):1387-1398.
131. Bernardo S, Marchionni E, Prudente S, et al. Unusual association of SCN2A epileptic encephalopathy with severe cortical dysplasia detected by prenatal MRI. Eur J Paediatr Neurol. 2017;21(3):587-590.
132. Liang JS, Lin LJ, Yang MT, Wang JS, Lu JF. The therapeutic implication of a novel SCN2A mutation associated early-onset epileptic encephalopathy with Rett-like features. Brain & development. 2017;39(10):877-881.
133. Trujillano D, Bertoli-Avella AM, Kumar Kandaswamy K, et al. Clinical exome sequencing: results from 2819 samples reflecting 1000 families. European journal of human genetics : EJHG. 2017;25(2):176-182.
134. Su DJ, Lu JF, Lin LJ, Liang JS, Hung KL. SCN2A mutation in an infant presenting with migrating focal seizures and infantile spasm responsive to a ketogenic diet. Brain & development. 2018;40(8):724-727.
135. Lauxmann S, Boutry-Kryza N, Rivier C, et al. An SCN2A mutation in a family with infantile seizures from Madagascar reveals an increased subthreshold Na(+) current. Epilepsia. 2013;54(9):e117-121.
136. Sahli M, Zrhidri A, Elaloui SC, et al. Clinical exome sequencing identifies two novel mutations of the SCN1A and SCN2A genes in Moroccan patients with epilepsy: a case series. J Med Case Rep. 2019;13(1):266-266.
